# Gut microbial dysbiosis, IgA, and *Enterococcus* in common variable immunodeficiency with immune dysregulation

**DOI:** 10.1186/s40168-024-01982-y

**Published:** 2025-01-16

**Authors:** Roos-Marijn Berbers, Fernanda L. Paganelli, Joris M. van Montfrans, Pauline M. Ellerbroek, Marco C. Viveen, Malbert R. C. Rogers, Moniek Salomons, Jaap Schuurmans, Martine van Stigt Thans, Remi M. M. Vanmaris, Lodewijk A. A. Brosens, Maria Marlot van der Wal, Virgil A. S. H. Dalm, P. Martin van Hagen, Annick A. J. M. van de Ven, Hae-Won Uh, Femke van Wijk, Rob J. L. Willems, Helen L. Leavis

**Affiliations:** 1 Department of Medical Microbiology, University Medical Center Utrecht and Utrecht University, Utrecht, the Netherlands; 2 Department of Rheumatology and Clinical Immunology, University Medical Center Utrecht and Utrecht University, Utrecht, the Netherlands; 3 Department of Pediatric Immunology and Infectious Diseases, University Medical Center Utrecht and Utrecht University, Utrecht, the Netherlands; 4 Department of Internal Medicine and Infectious Diseases, University Medical Center Utrecht and Utrecht University, Utrecht, the Netherlands; 5 Department of Pathology, University Medical Center Utrecht and Utrecht University, Utrecht, the Netherlands; 6 Center for Translational Immunology, University Medical Center Utrecht and Utrecht University, Utrecht, the Netherlands; 7 Department of Internal Medicine, Division of Clinical Immunology, Erasmus MC University Medical Center, Rotterdam, the Netherlands; 8 Department of Immunology, Academic Center for Rare Immunological Diseases (RIDC), Erasmus MC University Medical Center, Rotterdam, the Netherlands; 9 Departments of Internal Medicine and Allergology, Rheumatology and Clinical Immunology, University Medical Center Groningen, Groningen, the Netherlands; 10 Department of Data Science and Biostatistics, Julius Center, University Medical Center Utrecht and Utrecht University, Utrecht, the Netherlands

**Keywords:** Common variable immunodeficiency (CVID), Immune dysregulation, Gut microbiota, Pathobionts

## Abstract

**Background:**

Common variable immunodeficiency (CVID) is characterized by hypogammaglobulinemia and recurrent infections. Significant morbidity and mortality are caused by immune dysregulation complications (CVIDid), which affect around one-third of CVID patients and have a poorly understood etiology. Here, we investigate the hypothesis that gut microbial dysbiosis contributes to the inflammation underlying CVIDid.

**Results:**

Bacterial invasion of colonic crypts was observed in CVID (3/15) and X-linked agammaglobulinemia (XLA, 1/3), but not in healthy control (HC, 0/9) biopsies. Fecal gut microbiota was characterized using 16S rRNA-targeted amplicon sequencing. Increased bacterial load, decreased alpha diversity and distinct beta diversity were observed in CVIDid (*n* = 42) compared to HC (*n* = 48), and similar results were seen in CVID with IgA deficiency (*n* = 40) compared to HC. CVIDid and CVID-IgA showed enrichment of the genus *Enterococcus*, and in vitro studies confirmed the inflammatory potential of *Enterococcus gallinarum* and *Enterococcus hirae* in patient monocytes.

**Conclusions:**

This study further supports the hypothesis that a dysregulated gut microbiota, with IgA deficiency as an important driving factor, contributes to systemic inflammation in primary antibody deficiency, and introduces enterococci as potential pathobionts in CVIDid.

Video Abstract

**Supplementary Information:**

The online version contains supplementary material available at 10.1186/s40168-024-01982-y.

## Background

Common variable immunodeficiency disorder (CVID) is a primary immunodeficiency hallmarked by low serum immunoglobulins and impaired production of specific antibodies, resulting in an increased risk for infections with polysaccharide encapsulated bacteria [1, 2]. While the infection frequency and severity can be ameliorated with adequate immunoglobulin replacement therapy (IgRT), over a third of CVID patients still develop additional immune dysregulation complications such as autoimmune disease, granuloma formation, enteropathy, and lymphoproliferative disease with an increased risk of lymphoma [3, 4]. This CVID with immune dysregulation (CVIDid) phenotype causes significant morbidity and mortality, resulting in poorer long-term survival compared to CVID with infections only (CVIDio) [4–7]. The cause of immune dysregulation complications in CVID is not completely understood, and it has been hypothesized that dysbiotic gut microbiota may play a role in causing inflammation and clinical complications in CVIDid [8, 9]. A more profound antibody deficiency is observed in patients with X-linked agammaglobulinemia (XLA), which is caused by mutations in the early B cell development gene Bruton’s tyrosine kinase, and results in the complete absence of B-cells and immunoglobulins [10]. XLA patients typically do not show the same predisposition for the development of immune dysregulation complications as CVID patients [11].

The gut microbiota plays an important role in the maintenance of immune homeostasis and has been implicated in the pathogenesis of several autoimmune diseases [12]. The presence of commensal gut bacteria with a (suspected) causal link to the onset of disease—so-called pathobionts—is thought to contribute to diseases such as rheumatoid arthritis [13], systemic lupus erythematosus (SLE) and autoimmune hepatitis [14], and mouse models of multiple sclerosis [15]. One proposed mechanism by which gut commensals can drive an immune response to autoantigens is through molecular mimicry—if the gut commensal provides an antigenic stimulus that causes cross-recognition of host proteins by the adaptive immune system [16]. Alternatively, pathobionts may provide a broad pro-inflammatory stimulus that causes innate immune activation and contributes to autoimmunity in individuals who are susceptible to self-recognition [14].

The composition of the microbiota is partly regulated by the immune system, with an important role for immunoglobulin A (IgA) in the regulation of the microbiota at mucosal surfaces [17]. IgA is thought to not only influence the composition but also the localization of the microbiota, thereby limiting contact between the immune system and the microbiota [18]. IgA deficiency is a known risk factor for the development of immune dysregulation in CVID [19, 20], and is associated with more profound alterations in gut microbiota composition compared to CVID patients with normal IgA levels [21, 22]. These findings support the hypothesis that insufficient IgA may affect mucosal homeostasis, leading to an altered gut microbiota composition and translocation of microbial products in CVID, thereby contributing to inflammation in CVIDid. Differences in microbiota composition between CVIDid and CVIDio, however, remain poorly understood as most published CVID microbiota studies contain a mix of the two phenotypes and have low power to compare the two [21–23].

In the present study, we assessed the localization of the microbiota in gut biopsies of CVID and XLA patients, and we characterized the bacterial load and composition of the gut microbiota in CVIDio and CVIDid in a cross-sectional multicenter study. We finally relate the presence of pathobiont bacteria, in particular *Enterococcus* species, to inflammation markers in patient serum and assess their in vitro immunostimulatory capacities.

## Methods

### Ethics statement

Ethical approval for this study for all Dutch participants was received from the Medical Ethical Committee of the Erasmus MC University Medical Center in Rotterdam, the Netherlands (METC: 2013–026). Written informed consent was obtained from all patients and controls according to the Declaration of Helsinki.

### Study population

Patients aged seven or older diagnosed with CVID according to the criteria of the European Society for Immunodeficiencies criteria [1] were included during outpatient clinic visits of the University Medical Center Utrecht, the Erasmus MC University Medical Center Rotterdam, and the University Medical Center Groningen, in the Netherlands. Household members of patients were recruited as healthy controls (HC). Medication use was recorded up to 3 months prior to sampling. All CVID and XLA patients received IgRT at the time of sampling, with target IgG trough levels of > 7.0 g/L. Clinical characteristics were collected from electronic patient files.

### Biopsies

Residual biopsy tissue from endoscopic screening for gastrointestinal malignancy in patients with primary antibody deficiency was obtained from the Pathology Biobank of the UMC Utrecht and permission was granted by the UMC Utrecht Biobank Research Ethics Committee (TCBio 16–493). Biopsies from a control group of patients were age- and gender-matched to biopsies from CVID patients and only included if they had no histological abnormalities and the indication for the biopsy was unrelated to a (suspected) inflammatory or immunodeficient condition. All biopsies were formalin-fixed and paraffin-embedded. Slides of 4 µm were deparaffinated and stained with hematoxylin and eosin (H&E) for histopathological assessment, and used for immunofluorescence or fluorescence in situ hybridization (FISH) staining. All biopsies used in this study were also evaluated by a gastrointestinal pathologist of the UMC Utrecht in the context of regular care.

### Fluorescence in situ hybridization

Tissue slides were de-waxed and hybridized for 2 h at 50 °C with a universal bacterial EUB-338 probe (Cy3 ~ 5′-GCTGCCTCCCGTAGGAGT-3′ ~ Cy3, IDT DNA technologies) or a non-EUB control probe (Cy3 ~ 5′-ACT CCT ACG GGA GGC AGC-3′ ~ Cy3, IDT DNA technologies) in hybridization buffer (0.9 M NaCl, 20 mM TRIS at pH 7.5, SDS 0.1% wt/vol) with 20% formamide. Biopsies were analyzed with a confocal laser scanning microscope (CLSM, Leica SP5) at × 63 magnification with digital zoom for close-up images, using LAS AF software (Leica).

### Immunofluorescence staining

IgA staining was performed as described previously by Hendrickx et al. [24]. Briefly, after de-waxing and antigen retrieval (boiling in Na-citrate buffer pH 6 for 20 min), staining was performed with primary antibody against IgA (1:100 unlabeled goat anti-human IgA, Southern Biotech, USA) and secondary antibody (1:500 Alexa Fluor 488 donkey anti-goat IgG, Invitrogen/Thermo Fisher Scientific, USA). TO-PRO-3 iodide (1:1000 Molecular Probes/Life Technologies, the Netherlands) was added as a nuclear stain. Biopsies were analyzed with a confocal laser scanning microscope (CLSM, Leica SP5), using LAS AF software (Leica) at × 40 magnification, with digital zoom for close-up images.

### Histology

Hematoxylin and eosin-stained slides were scanned and stored at × 40 magnification (Hamamatsu Digital slide scanner, Japan), and assessed by two independent researchers using NDP.view2 software (Hamamatsu, Japan). For each transverse colon biopsy, 5 longitudinal crypts not in the proximity of a Peyer’s patch were chosen. For each crypt, the theca area of the eight largest goblet cells was measured and the length of the crypt was measured. Goblet cell theca area was used as a measure of the mucus productive capacity of these cells [25]. Biopsies that did not contain 5 suitable longitudinally cut crypts were excluded. The means of the two independent measurements were used for the final assessment.

### Serum measurements

Serum was collected at the time of fecal sampling, and stored at – 80 °C until analysis. IgA was measured in serum using a PEG-enhanced immunoturbidimetric method (Atellica CH, Siemens). Very low IgA was defined as serum IgA < 0.1 g/L, in order to be consistent with the first gut microbiota study in CVID by Jørgensen et al. [9]. Serum cytokines were measured using Olink proximity extension assays (immune response and inflammation panels), as reported previously [26].

### Fecal sample collection and DNA isolation

Fecal samples were stored at − 80 °C within 24 h of production. Total DNA from feces samples was isolated using the QiaAmp DNA stool mini kit (Qiagen) according to the manufacturer’s protocol with the addition of a bead-beating step with 0.1 mm zirconium beads in the stool lysis buffer (ASL buffer). DNA was stored at − 20 °C prior to further analysis.

### Bacterial load and species-specific qPCR

All primers and probes were ordered from IDT DNA technologies (Supplementary Table S1). qPCR was performed using a StepOnePlus RT-PCR system (ThermoFisher). Bacterial load in the fecal samples was estimated using the BactQuant qPCR, as described by Liu et al. [27]. 16S ribosomal RNA (rRNA) copy number calculations were performed using serial dilutions of a plasmid containing the target sequence (IDT DNA technologies). For the design of the species-specific qPCR assay, strains originating from human stools were selected from the laboratory collection of the Department of Medical Microbiology, UMC Utrecht (for an overview of all bacterial strains used in this study, see Supplementary Table S2). All qPCR assays were tested for cross-reactivity against the other strains.

### 16S rRNA sequencing and bioinformatics

The 469 basepair V3 and V4 hypervariable regions of the 16S rRNA gene were amplified and sequenced using the Illumina MiSeq instrument and Reagent Kit v3 (600 cycle) according to Fadrosh et al. [28]. The resulting amplicon pool generated a total of 10.2 million read-pairs (sample median of 60.9 k read-pairs). The QIIME2 microbial community analysis pipeline (version 2018.8) [29] was used with DADA2 [30] for sequence variant detection (with default settings, except for –p-trunc-len-f 275 –p-trunc-len-r 260), and SILVA as 16S rRNA reference gene database (SILVA 132) [31]. Sequencing data has been made available on the European Nucleotide Archive under project code PRJEB44275. Samples with total reads below 1500 were excluded from all analyses.

### Shotgun metagenomic sequencing and bioinformatics

Shotgun metagenomic sequencing of fecal samples was performed with the same DNA extracted for use in 16S rRNA sequencing. Sequence libraries were prepared using the Nextera XT Kit (Illumina, San Diego, CA, USA) according to the manufacturer’s instructions, using 1 ng of total DNA input. Libraries were sequenced by the Utrecht Sequencing Facility on an Illumina NextSeq 500 system with a 300-cycle (2 × 150 bp) NextSeq 500/550 High-Output v2.5 Kit. Illumina sequencing data were quality-assessed and trimmed using Trim Galore (default settings) [32].

Shotgun metagenomic sequencing of fecal DNA yielded 550 million paired-end reads (sample median of 13 million reads). Taxonomic classification was performed using the OneCodex platform [33] with the OneCodex database. Trimmed and quality-checked whole genome sequencing data was used for de novo genome assembly (metaSPAdes [34] with default settings and additional flag –only-assembler) and annotation (Prokka [35] with default settings).

### Selective culturing and whole genome sequencing of bacterial strains

For selective culturing of enterococci, fecal samples were first cultured in Enterococcosel broth [36] at 37 °C overnight. Next, liquid culture was plated on Slanetz and Bartley agar (Merck Millipore) (for isolation of *E. gallinarum* with the addition of 4 µg/mL vancomycin) and cultured for 48 h at 37 °C. Individual bacterial colonies were identified at the species level using MALDI-TOF mass spectrometry and stored at − 80 °C until further use.

Whole genome sequencing of cultured *Enterococcus* strains was performed as follows. DNA was isolated using the Wizard Genomic DNA Isolation Kit (Promega) with the protocol for gram-positive bacteria. Sequence libraries for Illumina sequencing were prepared using the Nextera XT Kit (Illumina, San Diego, CA, USA) according to the manufacturer’s instructions. Libraries were sequenced by the Utrecht Sequencing Facility on an Illumina NextSeq 500 system with a 300-cycle (2 × 150 bp) NextSeq 500/550 Mid-Output v2.5 Kit. Trimmed and quality-checked whole genome sequencing data was used for de novo genome assembly (metaSPAdes [34] with default settings and additional flag –only-assembler) and annotation (Prokka [35] with default settings).

### Monocyte cocultures

Bacterial supernatants were cocultured with the patient or HC cells as described by the Zitvogel group [37], with some modifications. Whole blood was collected from patients at the same time as serum and fecal sampling, and peripheral blood mononuclear cells were isolated by ficoll-density centrifugation (GE Healthcare-Biosciences, AB), and frozen at – 180 °C until use. For monocyte-bacterial coculture, PBMCs were thawed, and monocytes were isolated using magnetic-activated cell sorting using CD14 + microbeads (Miltenyi Biotech). Per condition, 5000 monocytes were plated in 96 well-plates in Iscove’s modified Dulbecco’s medium (IMDM, ThermoFisher) with 5% glutamine 5% hepes, and 10% human AB serum. Bacteria were prepared by inoculating a 5 mL brain–heart infusion (BHI) medium with enterococci isolated from patient stools, or *E. coli* E783 in 5 mL lysogeny broth. After overnight culture at 37 °C, bacterial supernatants were filter sterilized at 0.2 µm. For each condition, 20 µL bacterial supernatant was added to the monocytes and incubated at 37 °C and 5% CO2. The next day, culture supernatants were harvested and frozen at − 20 °C until further use. Cytokine measurements of IL-6 and IL-10 in culture supernatants were performed using Luminex technology [38].

### Data analysis and statistical methods

All analyses were performed using R 3.2.0 [39]. Continuous variables were compared using the Mann–Whitney rank test. Categorical variables were compared using a two-tailed Fisher’s exact test. Diversity indices were calculated using the packages *phyloseq* and *vegan*. Principal Component Analysis (PCA) was performed using the *prcomp* function on hyperbolic arcsine (asinh) transformed data. PERMANOVA with correction for false discovery rate (FDR) was used to detect global community differences in PCA using the package *vegan*.

Differential abundance testing was performed using ANCOM-BC [40] with Benjamini–Hochberg FDR correction using an alpha of 0.05 as a threshold for significance. All ANCOM-BC analyses were corrected for age and sex using the following formula: bacterium ~ patient group + age + sex. Bacterial taxa with zero read in more than 90% of samples were excluded from the analysis. For subgroup analyses within the immune dysregulation group, structural zero detection was not applied due to the small sample size and increased risk of false positives.

## Results

### Bacterial crypt invasion, local IgA deficiency, and altered crypt architecture in CVID

**Fig. 1:**
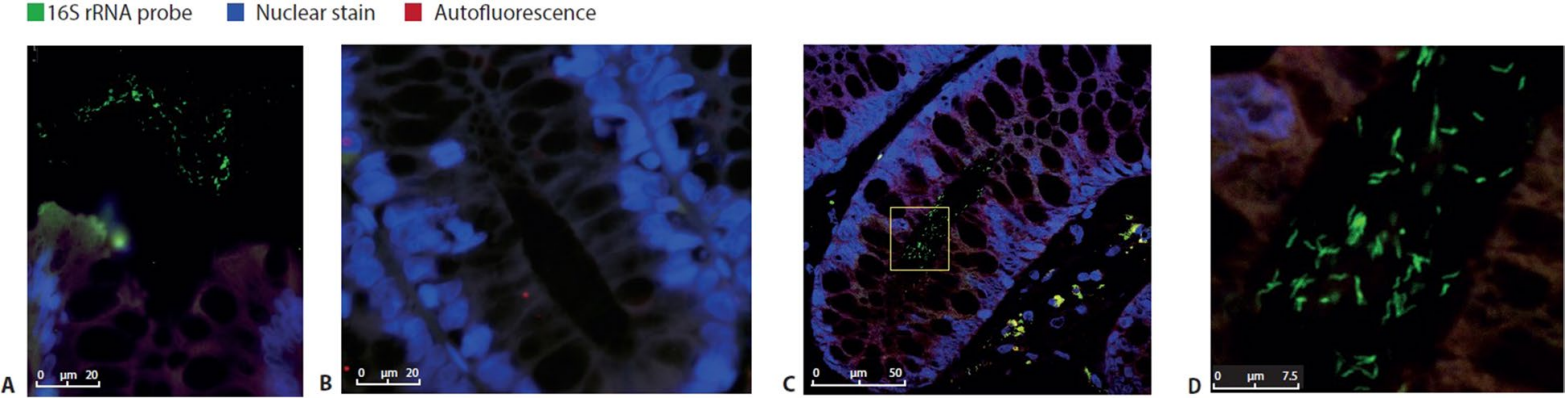
Localization of the microbiota in CVID. Representative 16S rRNA Fluorescence in situ hybridization (FISH) staining. Luminal microbiota (in green) were excited at 488 nm; DNA in the colon epithelium cell (in blue) was stained with DAPI at 405 nm. Images were taken using a Leica SP5 confocal microscope with a × 63 objective. **A** Luminal microbiota in a healthy control colon biopsy. **B** Colonic crypt without the presence of microbiota in a healthy control colon biopsy. **C** Representative 16S rRNA FISH staining showing bacterial presence in colonic crypts in a CVID patient. Yellow box indicates area enlarged in **D**. **D** Detail of **C**, showing microbiota invasion in colonic crypts of a CVID patient

In order to investigate the localization of the microbiota in primary antibody deficiency, we performed 16S rRNA FISH staining on colon biopsies from 15 CVID patients, 3 XLA patients, and 9 HC (Supplementary Table S3). No patients experienced clinical signs of enteritis at the time of sampling. As expected, luminal bacteria were detected in all biopsies (Fig. 1A). In 3/15 CVID patients and 1/3 XLA patients, but none of the 9 HC (Fig. 1B–D), the presence of bacteria deep inside colon crypts was detected. Of the CVID patients with crypt invasion, 2 had immune dysregulation complications (one had autoimmune gastritis, and the other had granulomatous-lymphocytic interstitial lung disease and splenomegaly). Crypt-invading bacteria were of mixed morphology, suggesting a location shift of a heterogeneous population of bacteria rather than of one invasive species (Fig. 1D).

**Fig. 2:**
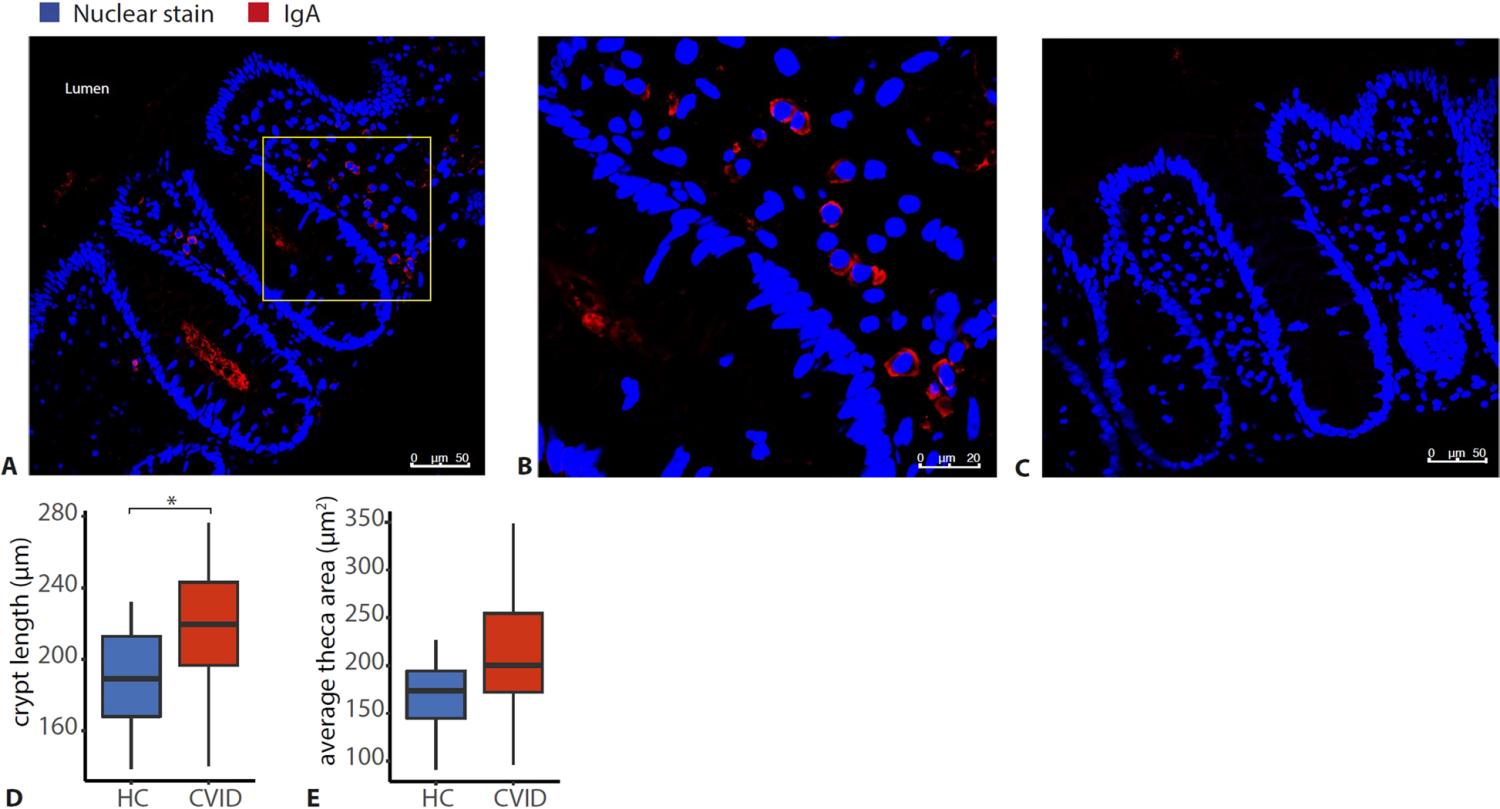
IgA deficiency and crypt architecture in CVID. **A** Representative IgA staining of a healthy control. Yellow box indicates area enlarged in **B**. IgA (in red) was excited at 488 nm; DNA in the colon epithelium cell (in blue) was stained with TO-PRO-3 iodide at 642 nm. Images were taken using a Leica SP5 confocal microscope with a × 40 objective. **B** Detail of **A** showing IgA + plasma cells, using digital zoom. **C** Representative IgA staining of a CVID patient with no detectable IgA in serum, and absence of IgA in colon biopsy. **D** Average crypt length in healthy control (HC, *n* = 9) and CVID (*n* = 15) colon biopsies. **E** Average Goblet cell theca area in healthy control (HC, *n* = 9) and CVID (*n* = 15) colon biopsies. The horizontal line inside the box represents the median. The whiskers represent the lowest and highest values within 1.5 × interquartile range. Statistics: Mann–Whitney *U* test. *p < 0.05, **p < 0.01, ***p < 0.001

As IgA is thought to contribute to the immune exclusion of the microbiota [18], IgA deficiency may contribute to the observed bacterial crypt invasion. Indeed, of the patients with crypt invasion, all had serum IgA < 0.1 g/L, and the biopsies showed lymphoid infiltration. To investigate whether serum IgA deficiency also reflects local IgA production in the gut in CVID, we stained the biopsies for secretory IgA. Indeed, in 4/4 CVID patients with serum IgA > 0.1 g/L and 9/9 HC, IgA + plasma cells were detected (Fig. 2A, B), while they were absent in 10/11 CVID patients with serum IgA < 0.1 g/L (Fig. 2C).

An additional explanation for bacterial crypt invasion could be related to the mucus layer, which is produced by Goblet cells in the gut and provides a physical barrier between the gut microbiota and the epithelium. Mucus thickness could not be directly investigated here due to the conservation method of the biopsies, but mucus deficiency is associated with crypt elongation and an increase in Goblet cell theca area in experimental models [41]. In our biopsies, we also observed crypt elongation (*p* = 0.019) (Fig. 2D) and a slight increase in the size of the Goblet cell theca area (Fig. 2E) in CVID that was not statistically significant (*p* = 0.101). Therefore, we conclude that disturbed IgA and mucus production may contribute to increased contact of bacteria with the epithelium in CVID.

### Increased fecal bacterial load in CVIDid without medication use

In order to investigate whether not only the location but also the abundance and composition of the gut microbiota is associated with inflammation in CVID, we collected fecal samples from 42 CVIDid, 51 CVIDio, 11 XLA patients, and 48 HC (Table 1). In this cohort, serum IgA was lower in CVIDid than CVIDio (*p* = 0.006), and normal (> 0.70 g/L) for all HC (Supplementary Fig. S1).

**Table 1:** Cohort overview

Summary statistics	HC (*N* = 48)	CVIDio (*N* = 51)	CVIDid (*N* = 42)	XLA (*N* = 11)
Characteristics				
Age-median (IQR)	43.50 (36.00, 49.50)	40 (25.00, 56.00)	40.50 (32.00, 53.75)	16 (12.00, 26.50)
Sex (male)	18 (37.50%)	22 (43.14%)	20 (47.62%)	11 (100.00%)
Center (Utrecht)	41 (85.42%)	30 (58.82%)	29 (69.05%)	9 (81.82%)
Antibiotics	0 (0.00%)	18 (35.29%)	20 (47.62%)	7 (63.64%)
Immunosuppressive medication	0 (0.00%)	5 (9.80%)	11 (26.19%)	1 (9.09%)
Serum IgA > 0.1 g/L	48 (100.00%)	36 (70.59%)	17 (40.48%)	0 (0.00%)
Immune dysregulation complications				
Pulmonary	0 (0.00%)	0 (0.00%)	11 (26.19%)	0 (0.00%)
Hematological	0 (0.00%)	0 (0.00%)	10 (23.81%)	0 (0.00%)
Gastrointestinal	0 (0.00%)	0 (0.00%)	22 (52.38%)	0 (0.00%)
Rheumatological	0 (0.00%)	0 (0.00%)	10 (23.81%)	0 (0.00%)
Dermatological	0 (0.00%)	0 (0.00%)	7 (16.67%)	0 (0.00%)
Lymphoproliferative	0 (0.00%)	0 (0.00%)	18 (42.86%)	0 (0.00%)
Other	0 (0.00%)	0 (0.00%)	6 (14.29%)	0 (0.00%)
Genetics				
Not done	48 (100.00%)	48 (94.12%)	30 (71.43%)	7 (63.64%)
Nothing found	0 (0.00%)	2 (3.92%)	4 (9.52%)	0 (0.00%)
Only VUS found	0 (0.00%)	0 (0.00%)	5 (11.90%)	0 (0.00%)
Pathogenic mutations found	0 (0.00%)	1 (1.96%)^a^	4 (9.52%)^b^	4 (36.36%)

*HC* healthy control, *CVIDio* CVID with infections only, *CVIDid* CVID with immune dysregulation, *XLA* X-linked agammaglobulinemia, *IQR* interquartile range. *VUS* variant of unknown significance

^a^Pathogenic mutations: TACI

^b^Pathogenic mutations: 1 CTLA4 haploinsufficiency, 1 STAT1GoF, 1 PI3KR1, 1 TACI

**Fig. 3:**
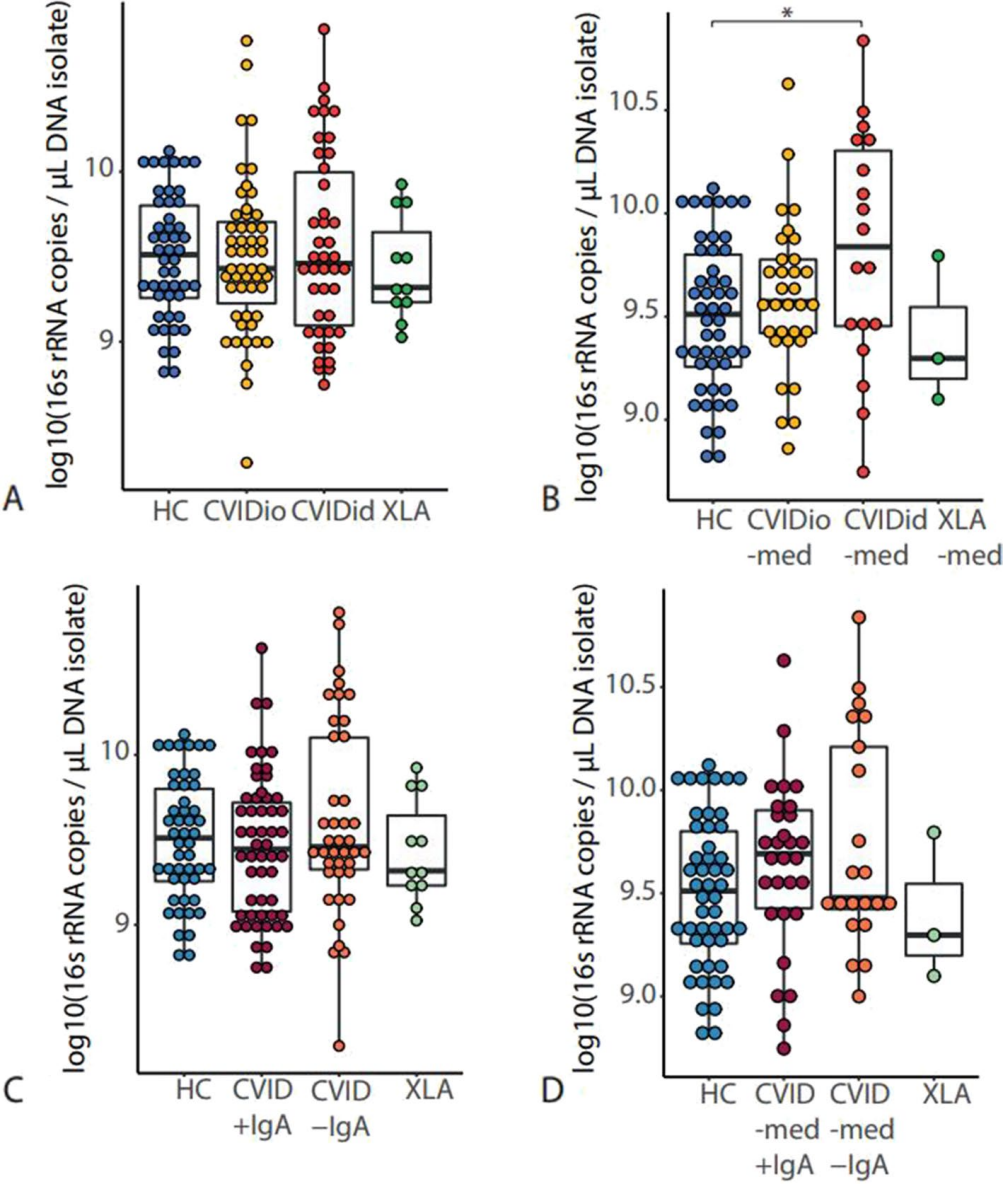
Bacterial load as determined using 16S rRNA qPCR in **A** healthy controls (HC *n* = 48), CVID with infections only (CVIDio, *n* = 51) CVID with immune dysregulation (CVIDid, *n* = 42), and X-linked agammaglobulinemia (XLA, *n* = 11). **B** Patients who did not use antibiotic or immunosuppressive treatment up to 3 months prior to sampling (-med). Healthy controls (HC *n* = 48), CVIDio-med (*n* = 32), CVIDid-med (*n* = 18) XLA-med (*n* = 3). **C** Healthy controls (HC, *n* = 48), CVID + IgA (*n* = 53), CVID-IgA (*n* = 40), XLA (*n* = 11). D: HC (*n* = 48), CVID –med + IgA (*n* = 29), CVID –med –IgA (*n* = 21), XLA–med (*n* = 3). The horizontal line inside the box represents the median. The whiskers represent the lowest and highest values within 1.5 × interquartile range. Statistics: Mann–Whitney *U* test. *p < 0.05, **p < 0.01, ***p < 0.001

The total amount of bacteria in each fecal sample was quantified by determining the 16S rRNA gene load using qPCR. There was no difference in bacterial load when all sampled patients were included (Fig. 3A), and we hypothesized that medication use may influence these results. Indeed, when patients who used antibiotics or immunosuppressive therapy 3 months prior to sampling were excluded, bacterial load was increased in CVIDid compared to HC (*p* = 0.02, Fig. 3B). In patients who did use medication, bacterial load was similar to that of HC (Supplementary Fig. S2). No difference in bacterial load between CVID with serum IgA < 0.1 g/L (CVID–IgA) and CVID with serum IgA > 0.1 g/L (CVID + IgA) was observed (Fig. 3C), regardless of medication use (Fig. 3D).

### Lower alpha diversity in CVIDid and CVID-IgA

**Fig. 4:**
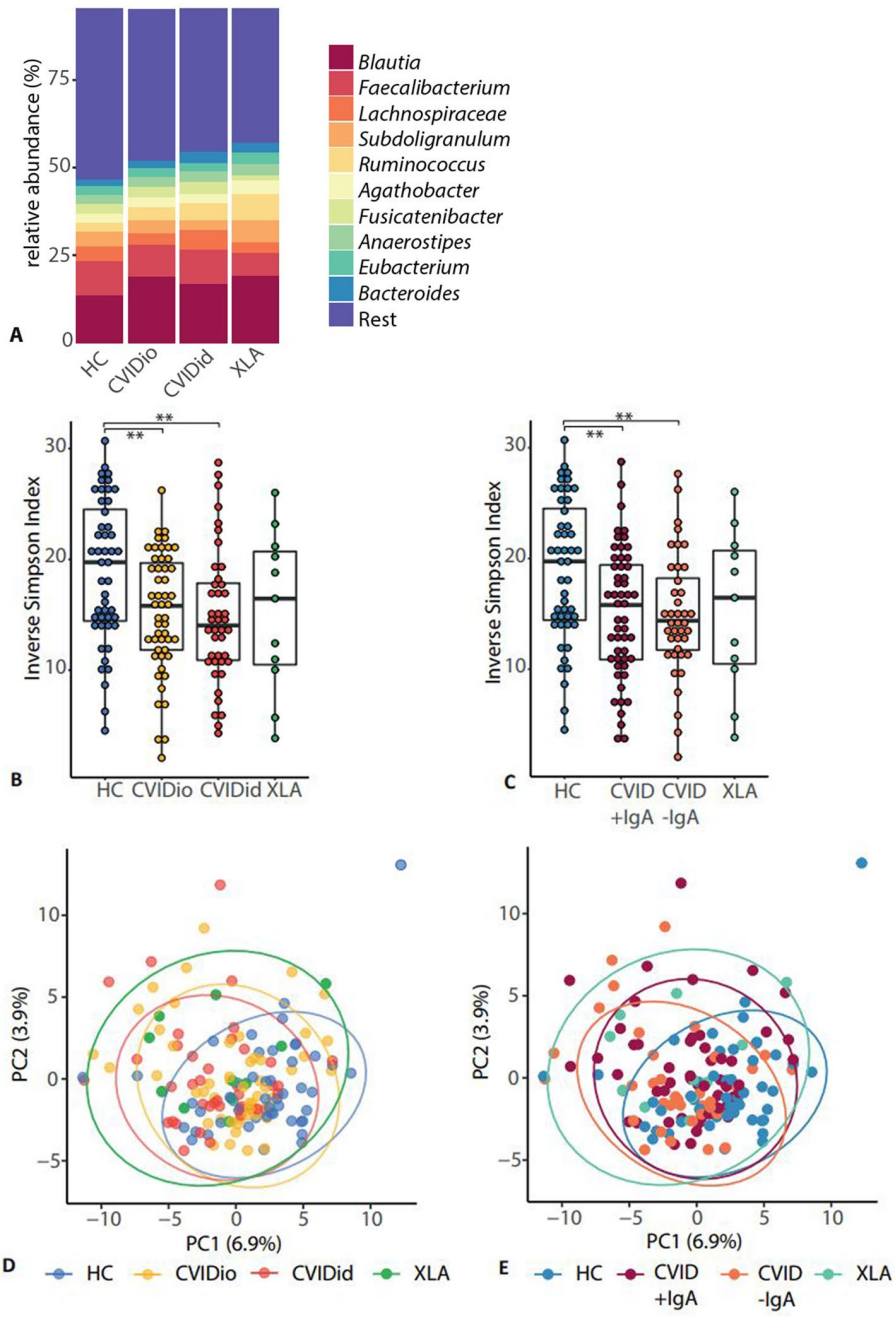
Composition of the gut microbiota in CVID as determined using 16S rRNA gene sequencing. Healthy controls (HC *n* = 48), CVID with infections only (CVIDio, *n* = 51) CVID with immune dysregulation (CVIDid, *n* = 42), X-linked agammaglobulinemia (XLA, *n* = 11), CVID + IgA (*n* = 53), CVID-IgA (*n* = 40). **A** Top 10 most abundant bacterial genera. **B** and **C** Alpha diversity as calculated using the inverse Simpson index. The horizontal line inside the box represents the median. The whiskers represent the lowest and highest values within 1.5 × interquartile range. Statistics boxplots: Mann–Whitney *U* test. *p < 0.05, **p < 0.01, *** *p* < 0.001. **D** and **E** Beta diversity is shown as principal component analysis (PCA) on genus-level hyperbolic arcsine transformed data. FDR-adjusted PERMANOVA: CVIDid vs HC adj. *p* = 0.006, CVIDio vs HC adj.* p* = 0.010. CVID + IgA vs HC adj. *p* = 0.006, CVID-IgA vs HC adj. *p* = 0.006. Ellipses indicate the 95% confidence interval

Taxonomic characterization of the fecal microbiota in CVID patients by 16S rRNA amplicon sequencing showed that the overall most abundant bacterial genera in feces (Fig. 4A) were *Blautia*, *Faecalibacterium,* and a further unidentified genus belonging to the Lachnospiraceae family. Microbial alpha diversity as expressed by inverse Simpson Index (Fig. 4B) was decreased in CVIDid compared to HC (*p* = 0.002), with CVIDio being the intermediate group (CVIDio vs HC *p* = 0.004). Alpha diversity was also decreased in the CVID-IgA group compared to HC (Fig. 4C, *p* = 0.002), with CVID + IgA as the intermediate group here (CVID + IgA vs HC *p* = 0.004). For both comparisons, similar trends were observed in the no-medication subanalysis, although not statistically significant (Supplementary Fig. S3A and B). Using a richness measure (Chao1) to evaluate alpha diversity also yielded similar results (Supplementary Fig. S4).

Beta diversity showed a small shift of both CVID phenotypes away from the HC (Fig. 4D; CVIDio vs HC *p*. adj = 0.010, CVIDid vs HC *p*. adj = 0.006), but no differences were observed within the CVID group, also not when split according to IgA status (Fig. 4E; CVID-IgA vs HC *p*. adj = 0.006, CVID + IgA vs HC *p*. adj = 0.006). Again, similar trends were observed in the no-medication subgroups, although not statistically significant (Supplementary Fig. S3D and E).

### *Bacteria* from the genus Enterococcus are associated with CVIDid and CVID-IgA

**Fig. 5:**
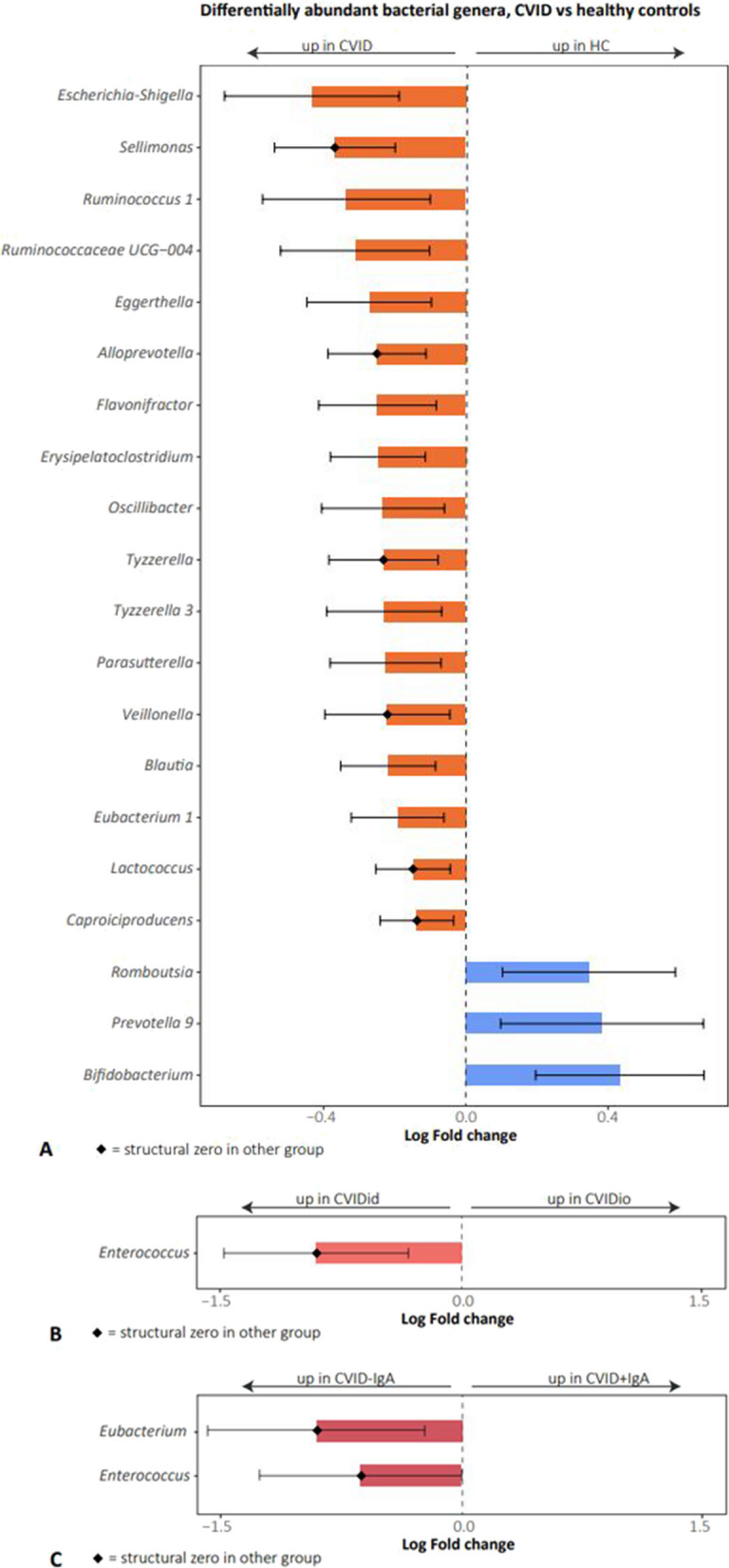
Differentially abundant bacterial genera as analyzed using ANCOM-BC. On the *X*-axis: log fold change. Bars represent confidence intervals. Diamond shape indicates structural zero in the other group. Only statistically significant (adjusted *p*-value < 0.05) results are displayed. **A** CVID (*n* = 93) versus healthy controls (HC, *n* = 48). **B** CVID with immune dysregulation (CVIDid, *n* = 42) versus CVID with infections only (CVIDio, *n* = 51). **C** CVID-IgA (*n* = 40) versus CVID + IgA (*n* = 53)

Next, we studied the distribution of bacterial taxa between all CVID patients and HC (Fig. 5A and Supplementary Table S4). Bacterial phyla Firmicutes (adj. *p* = 0.002) and Proteobacteria (adj. *p* = 0.004) were slightly more abundant in CVID compared to HC, while the class of Actinobacteria (adj. *p* = 0.048) was increased in HC. The bacterial group *Escherichia-Shigella* and its corresponding higher taxonomic levels showed the strongest association with CVID (effect size β − 0.433; standard error SE 0.125; adj. *p* = 0.011). *Escherichia-Shigella* was more often present in CVID (% zero in HC 62.50 vs 43.01 in CVID) and at higher abundance (0.22% in HC vs 0.47% in CVID). Other bacterial genera more abundant in CVID patients compared to HC were *Eggerthella, Alloprevotella, Lactococcus, Erysipelatoclostridium, Veillonella, Parasutterella*, and 11 bacteria belonging to the order Clostridiales: *Ruminococcus*, *Blautia*, *Sellimonas*, *Tyzzerella*, *Caproiciproducens*, *Flavonifractor*, *Oscillibacter* and a genus from the Ruminococcaceae UCG-004 group. When excluding patients with recent medication use, *Lactococcus, Veillonella, Sellimonas*, and *Tyzzerella* remained significantly more abundant in CVID (Supplementary Table S5).

Comparison of the composition of the fecal microbiota between CVIDid and CVIDio patients revealed that *Enterococcus* was exclusively detected in the feces of CVIDid patients and absent in the feces of CVIDio patients (adj. *p* = 0.000, Fig. 5B, Supplementary Table S6), also after exclusion of patients with recent antibiotic use (Supplementary Table S7). Enterococci could be detected in 10 out of 42 CVIDid patients. When comparing CVID + IgA to CVID-IgA, *Enterococcus* (adj. *p* = 0.000) and *Eubacterium* (adj. *p* = 0.000) were only detected in CVID-IgA and absent in CVID + IgA (Fig. 5C, Supplementary Table S8 and for the no medication analysis Supplementary Table S9).

### *Enterococcus* species as differentially abundant pathobionts in immune dysregulation in CVID

Since the presence of bacterial species from the genus *Enterococcus* has previously been associated with inflammation [42, 43] and the pathophysiology of autoimmune disease [14, 44], we next assessed whether the enterococci present in our cohort play a similar role in CVIDid. In order to investigate which species within the *Enterococcus* genus were present in the fecal flora of CVID patients, and might therefore be relevant to investigate further, shotgun metagenomic sequencing of 4 HC, 4 CVIDio, and 4 CVIDid was performed (Supplementary Fig. S5). Overall, the most frequently detected Enterococci were *Enterococcus casseliflavus, Enterococcus durans*, *Enterococcus faecium*, *Enterococcus faecalis*, *Enterococcus gallinarum*, and *Enterococcus hirae* (Supplementary Fig. S5). Of these, *E. casseliflavus* and *E. durans* were mostly detected in HC and not CVID, and therefore was excluded from further analysis.

**Fig. 6:**
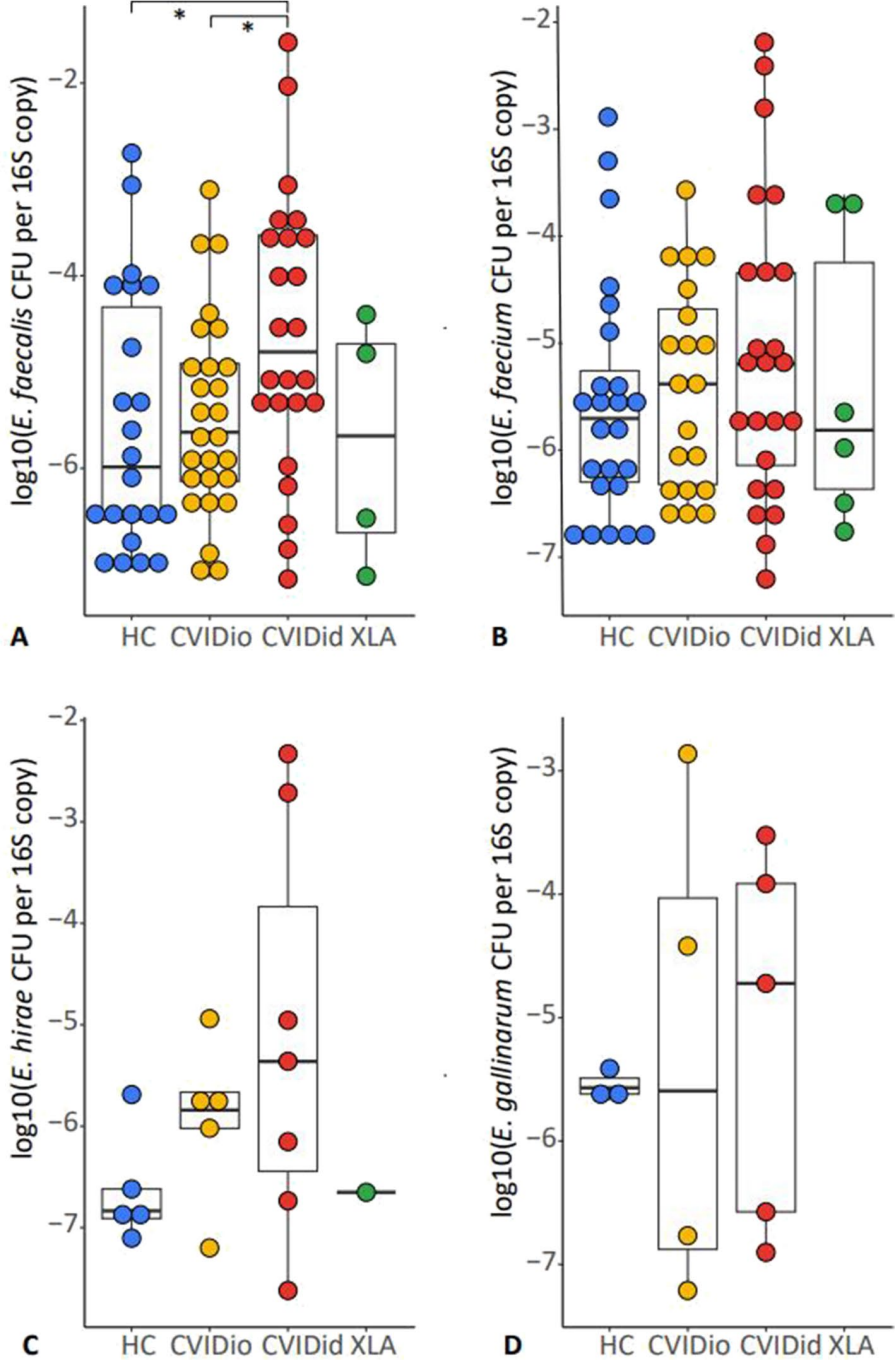
Presence of enterococcal DNA in stools as expressed by log 10 of the amount of enterococcal DNA present in one colony forming unit (CFU) corrected by the total 16S rRNA abundance per fecal sample as determined using qPCR. Results below the detection limit of the qPCR assay were not displayed. Healthy controls (HC *n* = 48), CVID with infections only (CVIDio, *n* = 51) CVID with immune dysregulation (CVIDid, *n* = 42), and X-linked agammaglobulinemia (XLA, *n* = 11). The horizontal line inside the box represents the median. The whiskers represent the lowest and highest values within 1.5 × interquartile range. Statistics: Mann–Whitney *U* test. *p < 0.05, **p < 0.01, ***p < 0.001

To confirm the presence of these specific enterococcal species in the whole cohort, species-specific qPCR assays were developed for *E. faecium*, *E. faecalis*, *E. gallinarum*, and *E. hirae*, and tested against the other species to ascertain that no cross-reactivity occurred (data not shown). The qPCR assay proved much more sensitive to detecting the presence of enterococcal DNA than 16S rRNA sequencing, and enterococcal DNA could be detected in the majority of stools, including HC and CVIDio samples. For all four enterococcal species tested, there was an overall trend of higher abundance in CVIDid than HC, with CVIDio as the intermediate group, that was only statistically significant for *E. faecalis* (Fig. 6; *E. faecalis* CVIDid vs CVIDio *p* = 0.03, CVIDid vs HC *p* = 0.03). The overall presence of *E. hirae* and *E. gallinarum* was much lower for all patient groups compared to *E. faecalis* and *E. faecium*. These trends persisted in the group without medication use, with the exception of *E. faecium* (Supplementary Fig. S6).

### *Enterococcus* strains associated with inflammatory cytokines in vivo and in vitro

**Fig. 7:**
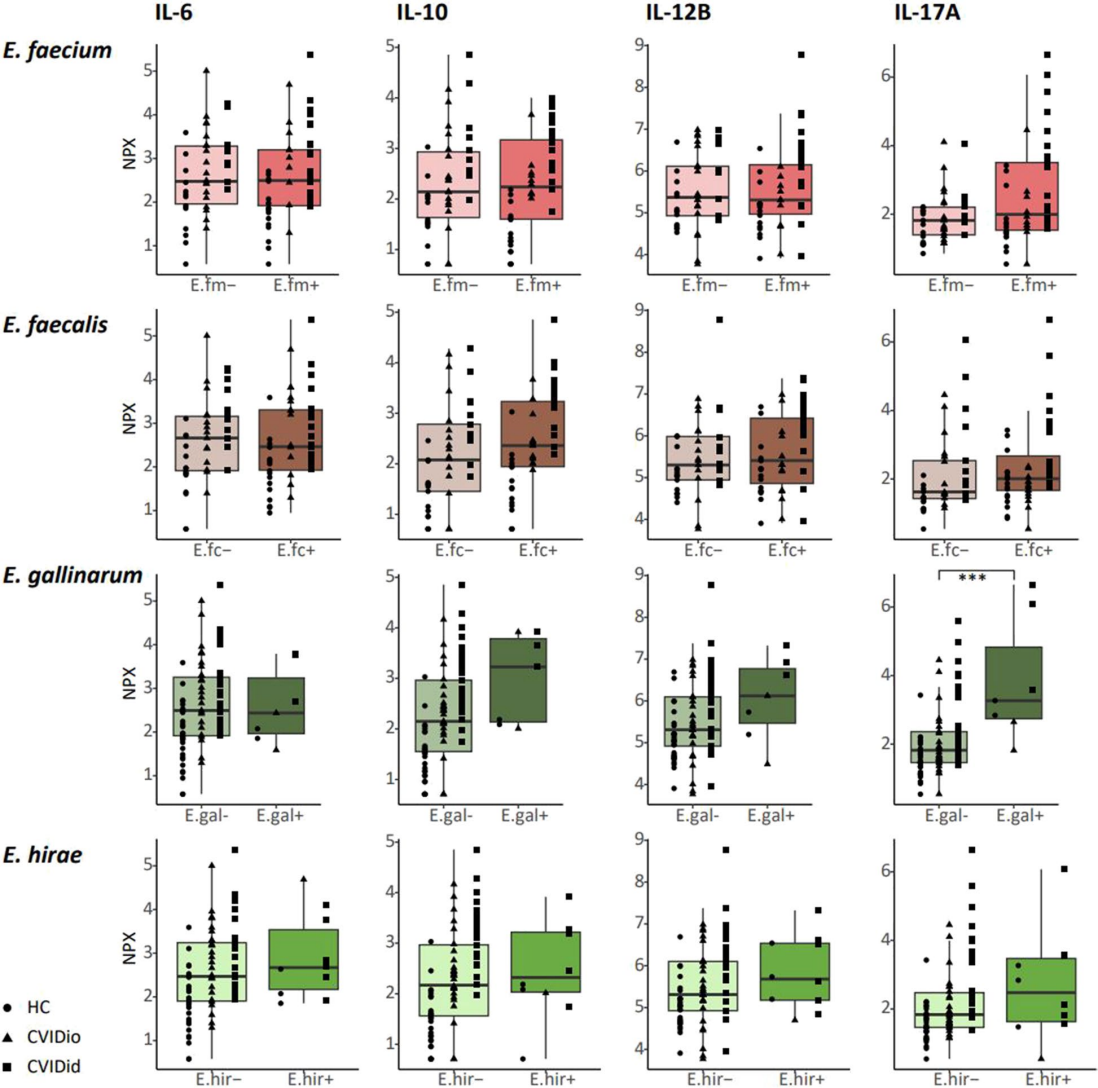
Serum cytokines as measured using targeted proteomics. NPX: normalized protein expression. The horizontal line inside the box represents the median. The whiskers represent the lowest and highest values within 1.5 × interquartile range. *p*-values: Mann–Whitney *U* test. *p < 0.05, **p < 0.01, ***p < 0.001

In order to investigate a potential link between carriage of enterococci and inflammation we questioned whether the presence of enterococci in stool was linked to cytokine levels in serum. To do this, we used serum cytokine levels as measured using Olink Proximity Extension analysis previously published by our group collected from the same cohort [26]. Not all samples could be perfectly matched, resulting in an overlapping cohort of 28 CVIDid patients, 29 CVIDio patients, and 31 healthy controls for which both cytokine and microbiome data were available, derived from serum and feces samples that were collected on the same day (Supplementary Table S10). Using this data, we compared levels of IL-6, IL-10, IL-12B, and IL-17A between individuals who did or did not have a positive qPCR for enterococci in their stools (Fig. 7).

Carriage of *E. gallinarum* (*n* = 2 CVIDid, *n* = 2 CVIDio, *n* = 3 HC with *E. gallinarum* vs *n* = 26 CVIDid, *n* = 27 CVIDio, *n* = 27 HC without *E. gallinarum*) was associated with increased serum levels of IL-17A (*p* = 0.004, Fig. 7), a pro-inflammatory cytokine produced by T-cells that is strongly associated with autoimmune diseases. None of the other enterococcus-cytokine combinations yielded statistically significant associations. We next tested the immunostimulatory capacity of *E. gallinarum* strains isolated from CVIDid patient stools in vitro, using *E. hirae* and *E. coli* strains for comparison. Bacterial supernatants from *E. gallinarum, E*. *hirae*, or *E. coli* were cocultured with primary monocytes isolated from CVIDid (*n* = 10), CVIDio (*n* = 10), or HC (*n* = 10). IL-6 and IL-10 were measured to reflect pro- or anti-inflammatory cytokine production by monocytes, respectively, as these cytokines were previously consistently found to be increased in CVID(id) in multiple cohorts [26, 45] and are produced by monocytes.

**Fig. 8:**
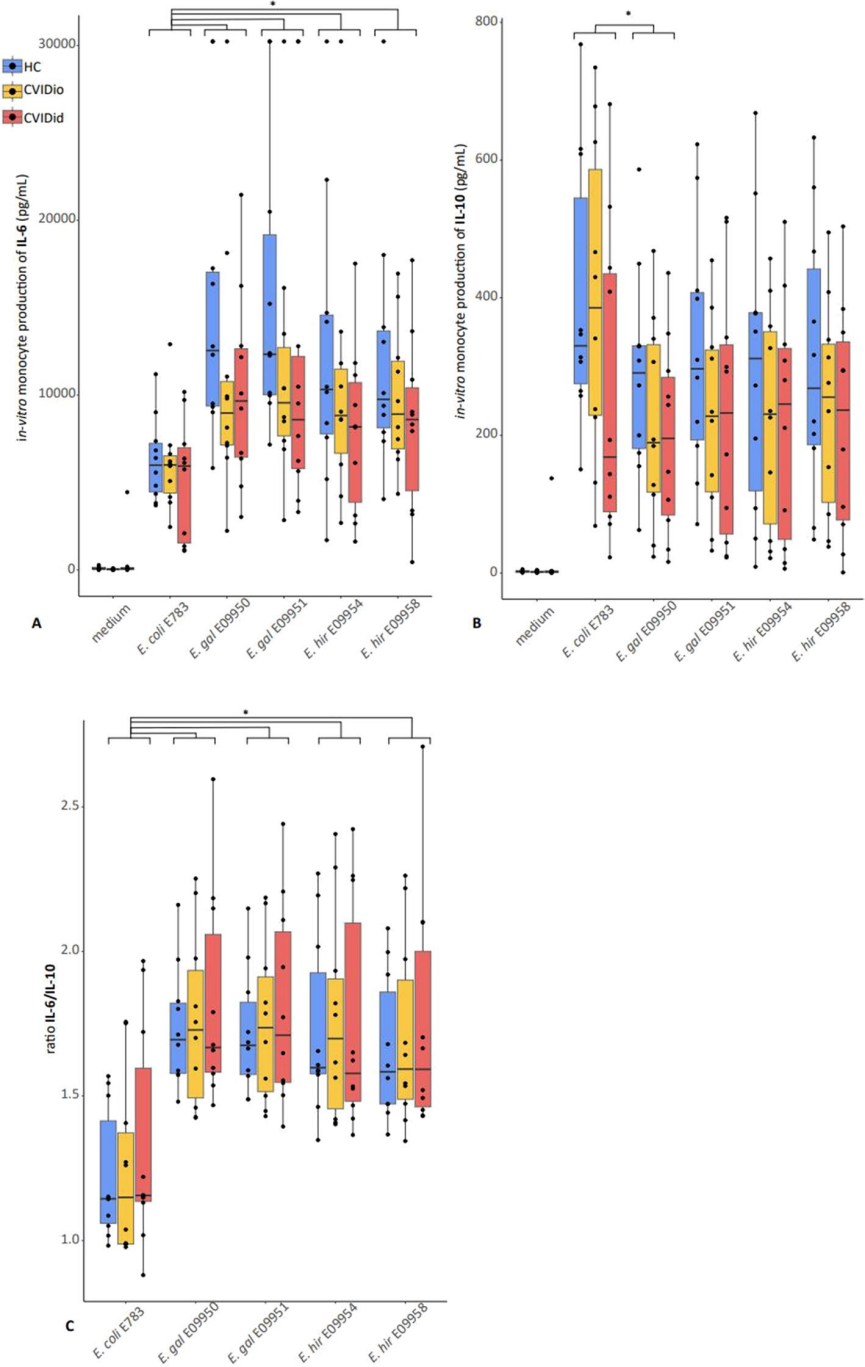
Cytokine production after *in-vitro* stimulation of monocytes from 10 healthy control (HC), 10 CVID with infections only (CVIDio) and 10 CVID with immune dysregulation (CVIDid) with bacterial supernatants from *E.coli* strain E783, *E.gallinarum* strain E9950, *E.gallinarum* strain E9951, *E.hirae* strain E9954 and *E.hirae* strain E9958. **A** IL-6 production, **B** IL-10 production, **C** IL6/IL10 ratio. The horizontal line inside the box represents the median. The whiskers represent the lowest and highest values within 1.5 × interquartile range. *P*-values: Mann–Whitney *U* test. *p < 0.05, **p < 0.01, ***p < 0.001

Compared to *E. coli*, stimulation with *E. gallinarum* and *E. hirae* consistently resulted in higher pro-inflammatory IL-6 production (*p* < 0.05 for all tested strains), and similar or slightly lower (for *E. gallinarum* strain E09950, *p* = 0.04) immune regulatory IL-10 production, regardless of whether the cells used were derived from HC, CVIDio or CVIDid patients (Fig. 8). Together, this resulted in a higher IL6/IL10 ratio for the *Enterococcus* strains compared to *E. coli* (Fig. 8C). No significant differences were observed between the *E. gallinarum* and the E. *hirae* strains. Overall, monocytes from CVIDid patients produced less IL-6 and IL-10 than HC groups when all tested bacteria were combined (*p* = 0.008 and *p* = 0.013 respectively, Supplementary Fig. S7), with CVIDio monocytes being the intermediate group. The ratio between IL6 and IL10 was not affected.

## Discussion

Recent microbiome studies have supported the presence of gut microbial dysbiosis in CVID patients [9, 23, 46, 47], but the role of specific gut bacteria in inflammation in CVID patients with immune dysregulation is thus far poorly understood. In this study, we demonstrated that increased contact of the gut microbiota with the colon epithelium occurs in some CVID patients and that the microbiota of CVIDid patients is characterized by an increased bacterial load, decreased alpha diversity, distinct beta diversity, and enrichment with enterococcal species. Interestingly, these findings on CVIDid versus CVIDio subgroups were highly similar to CVID-IgA versus CVID + IgA subgroups, while these patient groups are only 66% overlapping (36 CVIDio + IgA + 25 CVIDid-IgA / the total cohort of 93 CVID patients). Further analysis revealed that *E. gallinarum* was associated with a pro-inflammatory cytokine signal in the serum of patients and that *E. gallinarum* and *E. hirae* stimulated monocytes in vitro.

The bacterial crypt invasion we detected in some IgA-deficient CVID and XLA patients suggests that there is increased contact between the gut microbiota and the epithelium in these patients. A limitation of this experiment is the sample size of both the antibody deficient as the healthy control groups and the lack of quantification of bacteria. However, colonic crypts are generally accepted to be sterile under homeostatic conditions in healthy individuals (e.g., [48]), and therefore these findings are likely to be pathogenic. Moreover, biopsies only provide local snapshots of processes occurring in the gut, and therefore the true incidence of bacterial crypt invasion in CVID may be higher than we were able to observe here. Contact between the gut microbiota and the epithelium may trigger local inflammation [41], and contribute to systemic inflammation if it results in the previously reported endotoxemia in CVID(id) patients [9, 49–51]. Increased contact of the microbiota with the colonic epithelium has been reported in inflammatory bowel diseases [25] as well as colonic cancer [52], but to our knowledge, this has not previously been described in the context of antibody deficiency in CVID. In patients with antibody deficiency, IgA may play a role in maintaining sterility of the inner mucus layer. An association between mucosal IgA levels and CVID gut inflammation was previously reported [47], and mechanistic studies have provided examples of how interactions between IgA and gut commensals influence the localization of bacteria in the gut [53]. In children with IgA deficiency, a larger presence of microbiota-IgG was reported, and this was correlated with markers of systemic immune dysregulation [54], supporting our present hypothesis. The hypothesis that IgA deficiency in CVID leads to increased exposure to the gut microbiota warrants further investigation.

The histological findings in gut biopsies of CVID patients in our study were comparable to that observed in MUC2 deficient mice with crypt elongation and bacterial crypt invasion [41] and it is possible that defects in mucus production may also occur in CVID. Unfortunately, the fixation method of the biopsies available for this study was not suitable to directly investigate the mucus layer in order to further investigate that question.

Our 16S rRNA sequencing findings confirm and expand on previous studies investigating the CVID microbiome compared to HC. Decreased alpha diversity and distinct beta diversity have consistently been observed in CVID [9, 46, 55, 56], and these were more pronounced in both the CVIDid and the CVID-IgA subgroups. Interestingly, bacterial load was not altered in the CVID-IgA group while it was increased in CVIDid (when recent medication use was excluded). This implies that while IgA may impact the localization and the composition of the microbiota, other factors such as medication use have a larger impact on bacterial load than IgA deficiency.

The composition of the microbiota in CVID versus HC also matched earlier findings, including an association of decreased *Bifidobacterium* [9] and increased Enterobacteriaceae [9, 46] and *Eggerthella *[38] in CVID. Fiedorová et al. [46] also reported enrichment of *Enterococcus* in CVID, but did not specify whether this was more present in patients with immune dysregulation symptoms. Cabanero-Navalon et al. do not detect *Enterococcus* species as differentially abundant bacteria in CVID immune dysregulation. Differences in the detection of minority species such as enterococci between studies may be influenced by the specific DNA isolation protocol used, especially in the case of Gram-positives, which have a strong cell wall that is resistant to many lysis methods [57]. Also, regional microbiota differences between cohorts and increased statistical power due to a larger sample size are likely to influence differences between studies.

Our comparison of CVIDid to CVIDio and CVID-IgA to CVID + IgA showed an increased prevalence of *Enterococcus* species in the stools of CVIDid and CVID-IgA patients. In order to further investigate whether *Enterococcus* spp. could be implicated in the immune dysregulation in CVIDid patients, we showed the association of *E. gallinarum* with a pro-inflammatory cytokine signal in the serum of patients and showed the ability of *E. gallinarum* and *E. hirae* to stimulate monocytes in vitro. *E. gallinarum* has been previously linked with other immune dysregulation syndromes such as SLE [14], autoimmune hepatitis [14], and primary sclerosing cholangitis [44], while *E. hirae* has been linked to improved anti-cancer effects in patients receiving immune checkpoint inhibitor therapy, possibly due to its immunostimulatory effects [37]. Enteroccocci have been shown to be potential translocating bacteria in SLE and autoimmune hepatitis [14], as well as in an IgA-deficient mouse model [54]. As these enterococci are not exclusively present in CVID patients with immune dysregulation, they might present one of several contributing factors that together lead to the immune dysregulation phenotype.

One limitation of this study is the discrepancy between the 16S rRNA results and the species-specific qPCR. While the 16S sequencing results suggested a complete absence (or structural zero) of enterococci in the CVIDio and CVID + IgA groups, species-targeted qPCR yielded a far higher prevalence of *Enterococcus* species in the entire cohort, especially of the dominant taxa *E. faecalis* and *E. faecium*. Interestingly, one paper from 1990 already reports increased numbers of enterococci in the stools of patients with IgA deficiency and untreated CVID detected using quantitative culturing [58]. This illustrates the limited sensitivity of 16S rRNA high-throughput sequencing and stresses the importance of validating microbiota findings with targeted molecular techniques such as qPCR, and where possible, culturing when indexing minority species in the gut.

A second important limitation of this study is the cross-sectional study design, which allows for the investigation of correlation but not causation. The results of this study are therefore intended to be hypothesis-generating and suggest future areas of study, and cannot definitively point towards causes of immune dysregulation in CVID. Hopefully, future studies that follow patients longitudinally may shed more light on the causational relationship of IgA deficiency, carriage of specific microbiota signatures such as the prevalence of enterococci, and the development of clinical immune dysregulation.

## Conclusion

Our results support the hypothesis that in CVID, increased contact of the microbiota with the host epithelium and microbial dysbiosis contribute to immune dysregulation. These findings were consistent in patients with CVID and IgA deficiency. We hypothesize that microbial dysbiosis, possibly due to IgA deficiency and/or antibiotic use, may facilitate colonization of pathobionts such as enterococci and that sustained exposure to bacterial products may lead to innate immune system activation, which can provide an extra stimulus to an already autoimmunity-prone immune system [59]. Further studies are necessary to confirm the role of IgA deficiency and pathobionts such as *E. gallinarum* and *E. hirae* in the pathogenesis of immune dysregulation in CVID. Therapeutic targeting of gut pathobionts may be a promising future outlook in the prevention and treatment of immune dysregulation in CVID.

## Supplementary Information

Supplementary Material 1: Supplementary Figure S1. Serum IgA in healthy controls (HC, *n* = 48), CVID with infections only (CVIDio, *n* = 51) and CVID with immune dysregulation (CVIDio *n* = 42). The horizontal line inside the box represents the median. The whiskers represent the lowest and highest values within 1.5 × interquartile range. *P* values boxplots: Mann–Whitney U test. * *p* < 0.05, ** *p* < 0.01, *** *p* < 0.001. Supplementary Figure S2. Bacterial load as determined using 16S rRNA qPCR in: A: HC (*n* = 48), CVIDio –med (*n* = 32), CVIDio + med (*n* = 19), CVIDid –med (*n* = 18), CVIDid + med (*n* = 24), XLA (*n* = 11). HC: healthy control, CVIDio: CVID with infections only, CVIDid: CVID with immune dysregulation, XLA: X-linked agmmaglobulinemia. Med: patients who did ( +) or did not (-) use antibiotics or immunosuppressive therapy up to 3 months prior to sampling. The horizontal line inside the box represents the median. The whiskers represent the lowest and highest values within 1.5 × interquartile range. *P* values boxplots: Mann–Whitney U test. * *p* < 0.05, ** *p* < 0.01, *** *p* < 0.001. Supplementary Figure S3. Alpha diversity as calculated using the inverse Simpson index. The horizontal line inside the box represents the median. The whiskers represent the lowest and highest values within 1.5 × interquartile range. *P* values boxplots: Mann–Whitney U test. * *p* < 0.05, ** *p* < 0.01, *** *p* < 0.001. Supplementary Figure S4. Richness as calculated using the Chao1 index. The horizontal line inside the box represents the median. The whiskers represent the lowest and highest values within 1.5 × interquartile range. *P* values boxplots: Mann–Whitney U test. * *p* < 0.05, ** *p* < 0.01, *** *p* < 0.001. Supplementary Figure S5. Relative abundance of the most frequently detected enterococcal species determined using shotgun metagenomic sequencing in 4 HC, 4 CVIDio and 4 CVIDid. The horizontal line inside the box represents the median. The whiskers represent the lowest and highest values within 1.5 × interquartile range. Supplementary Figure S6. Presence of enterococcal DNA in stools of controls and patients *without medication use*. This is expressed by the log 10 of the amount of enterococcal DNA present in one colony forming unit (CFU) corrected by the total 16S rRNA abundance per fecal sample as determined using qPCR. Results below the detection limit of the qPCR assay were not displayed. HC (HC, *n* = 48), CVID with infections only (CVIDio, *n* = 32), CVID with immune dysregulation (CVIDid, *n* = 18), X-linked agammaglobulinemia (XLA, *n* = 3). Supplementary Figure S7. Cytokine production after *in-vitro* stimulation of monocytes from 10 HC, 10 CVIDio and 10 CVIDid with bacterial supernatants from *E.coli* E783, *E.gallinarum* E9950, *E.gallinarum* E.9951, *E.hirae* E9954 and *E.hirae* E9958 (pooled data). A: IL-6 production, B: IL-10 production, C: IL6/IL10 ratio. Supplementary Table S1. qPCR primer–probe sequences, ordered from IDT DNA technologies. All sequences reported from 5′-3’, all probes flanked by 6-FAM/ZEN (5’) and Iowa Black®Fluorescence Quencher^2^. Supplementary Table S2. Overview of all bacterial strains used in this study. Supplementary Table S3. Characteristics biopsy cohort. Supplementary Table S4. Differentially abundant bacteria identified using 16S rRNA sequencing. CVID (*n* = 93) vs healthy control (HC, *n* = 48): coefficients, standard errors (SE), *p*-values, FDR-adjusted *p*-values. Median relative abundance of non-zero counts per group. Statistics: ANCOM-BC. Supplementary Table S5. Differentially abundant bacteria identified using 16S rRNA sequencing. CVID without use of antibiotics or immunosuppressive therapy 3 months prior to sampling (CVID-med *n* = 50) vs healthy control (HC. *n* = 48): coefficients. standard errors (SE), *p*-values, FDR-adjusted *p*-values. Median relative abundance of non-zero counts per group. Statistics: ANCOM-BC. Supplementary Table S6. Differentially abundant bacteria identified using 16S rRNA sequencing. CVID with immune dysregulation (CVIDid. *n* = 42) versus CVID with infections only (CVIDio. n = 51): coefficients. standard errors (SE), *p*-values, FDR-adjusted *p*-values. Median relative abundance of non-zero counts per group. Statistics: ANCOM-BC. Supplementary Table S7. Differentially abundant bacteria identified using 16S rRNA sequencing. CVID with immune dysregulation without medication use (CVIDid -med. *n* = 18) versus CVID with infections only without medication use (CVIDio -med. *n* = 32). Patients did not use antibiotics or immunosuppressive therapy 3 months prior to sampling: coefficients. standard errors (SE), *p*-values, FDR-adjusted *p*-values. Median relative abundance of non-zero counts per group. Statistics: ANCOM-BC. Supplementary Table S8. Differentially abundant bacteria identified using 16S rRNA sequencing. CVID with IgA < 0.1 g/L (CVID-IgA *n* = 40) versus CVID with IgA > 0.1g/L (CVID + IgA *n* = 53): coefficients. standard errors (SE). *p*-values. FDR-adjusted *p*-values. Median relative abundance of non-zero counts per group. Statistics: ANCOM-BC. Supplementary Table S9. Differentially abundant bacteria identified using 16S rRNA sequencing. CVID with IgA < 0.1 g/L without medication use (CVID-IgA-med *n* = 21) versus CVID with IgA > 0.1g/L without medication use (CVID + IgA-med *n* = 29): coefficients. standard errors (SE). *p*-values. FDR-adjusted *p*-values. Median relative abundance of non-zero counts per group. Statistics: ANCOM-BC. Supplementary Table 10. Baseline characteristics of the matched serum-stool cohort.

## Data Availability

Sequencing data for this study has been made publicly available on the European Nucleotide Archive under project code PRJEB44275. Metadata can be found under the supplementary information section of this paper. R scripts can be found on Gitlab: https://gitlab.com/rberbers/cvid_mbiota_gut/.

## Abbreviations

CLSM: Confocal laser scanning microscope
CVID: Common variable immunodeficiency
CVIDid: CVID with immune dysregulation
CVIDio: CVID with infections only
FISH: Fluorescence in situ hybridization
H&E: Hematoxylin and eosin
HC: Healthy controls
IgA: Immunoglobulin A
IgRT: Immunoglobulin replacement therapy
LPS: Lipopolysaccharide
rRNA: Ribosomal RNA
SLE: Systemic lupus erythematosus
XLA: X-linked agammaglobulinemia
