# Supplementary material for: Gut microbial dysbiosis, IgA, and Enterococcus in common variable immunodeficiency with immune dysregulation
Source: Microbiome. 2025 Jan 16;13:12. doi: 10.1186/s40168-024-01982-y (PMC11740714; doi:10.1186/s40168-024-01982-y)
Supplement: Supplementary file 2 — Supplementary Material 1: Supplementary Figure S1. Serum IgA in healthy controls (HC, n = 48), CVID with infections only (CVIDio, n = 51) and CVID with immune dysregulation (CVIDio n = 42). The horizontal line inside the box represents the median. The whiskers represent the lowest and highest values within 1.5 × interquartile range. P values boxplots: Mann–Whitney U test. * p < 0.05, ** p < 0.01, *** p < 0.001. Supplementary Figure S2. Bacterial load as determined using 16S rRNA qPCR in: A: HC (n = 48), CVIDio –med (n = 32), CVIDio + med (n = 19), CVIDid –med (n = 18), CVIDid + med (n = 24), XLA (n = 11). HC: healthy control, CVIDio: CVID with infections only, CVIDid: CVID with immune dysregulation, XLA: X-linked agmmaglobulinemia. Med: patients who did ( +) or did not (-) use antibiotics or immunosuppressive therapy up to 3 months prior to sampling. The horizontal line inside the box represents the median. The whiskers represent the lowest and highest values within 1.5 × interquartile range. P values boxplots: Mann–Whitney U test. * p < 0.05, ** p < 0.01, *** p < 0.001. Supplementary Figure S3. Alpha diversity as calculated using the inverse Simpson index. The horizontal line inside the box represents the median. The whiskers represent the lowest and highest values within 1.5 × interquartile range. P values boxplots: Mann–Whitney U test. * p < 0.05, ** p < 0.01, *** p < 0.001. Supplementary Figure S4. Richness as calculated using the Chao1 index. The horizontal line inside the box represents the median. The whiskers represent the lowest and highest values within 1.5 × interquartile range. P values boxplots: Mann–Whitney U test. * p < 0.05, ** p < 0.01, *** p < 0.001. Supplementary Figure S5. Relative abundance of the most frequently detected enterococcal species determined using shotgun metagenomic sequencing in 4 HC, 4 CVIDio and 4 CVIDid. The horizontal line inside the box represents the median. The whiskers represent the lowest and highest values within 1.5 × in [file 40168_2024_1982_MOESM1_ESM.docx]

Supplementary Figures


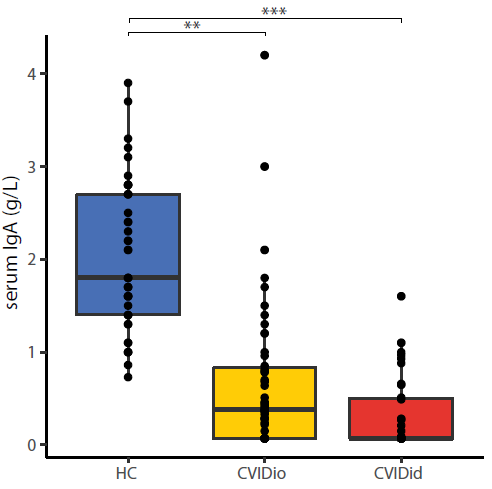


**Supplementary Figure 1A**: serum IgA in healthy controls (HC, n=48), CVID with infections only (CVIDio, n=51) and CVID with immune dysregulation (CVIDio n=42). The horizontal line inside the box represents the median. The whiskers represent the lowest and highest values within 1.5 × interquartile range. P values boxplots: Mann–Whitney U test. * p<0.05, ** p<0.01, *** p<0.001.


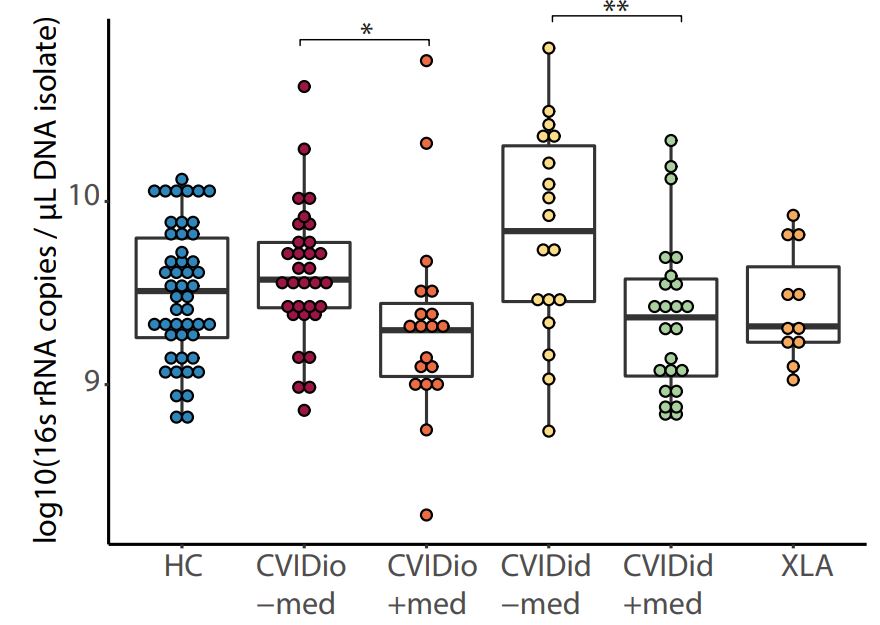


**Supplementary Figure 2**: Bacterial load as determined using 16S rRNA qPCR in:

A: HC (n=48), CVIDio –med (n=32), CVIDio +med (n=19), CVIDid –med (n=18), CVIDid +med (n=24), XLA (n=11).

HC: healthy control, CVIDio: CVID with infections only, CVIDid: CVID with immune dysregulation, XLA: X-linked agmmaglobulinemia. Med: patients who did (+) or did not (-) use antibiotics or immunosuppressive therapy up to 3 months prior to sampling. The horizontal line inside the box represents the median. The whiskers represent the lowest and highest values within 1.5 × interquartile range. P values boxplots: Mann–Whitney U test. * p<0.05, ** p<0.01, *** p<0.001.


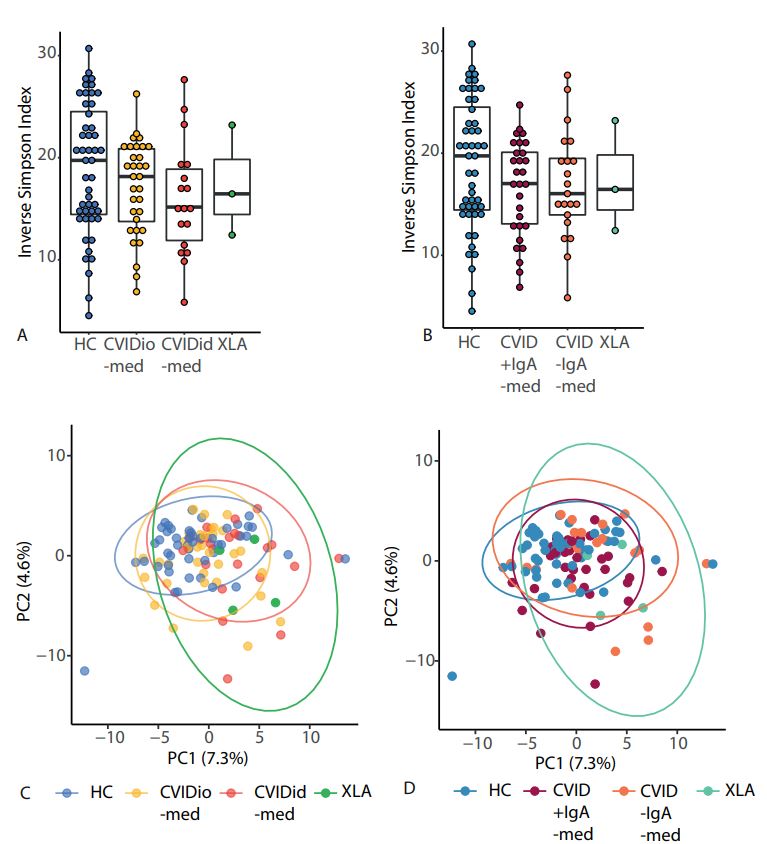


**Supplementary Figure 3:**

Alpha diversity as calculated using the inverse Simpson index. The horizontal line inside the box represents the median. The whiskers represent the lowest and highest values within 1.5 × interquartile range. P values boxplots: Mann–Whitney U test. * p<0.05, ** p<0.01, *** p<0.001.

A: HC (n=48), CVIDio –med (n=32), CVIDid –med (n=18), XLA-med (n=3).

B: HC (n=48), CVID –med +IgA (n=29), CVID –med –IgA (n=21), XLA –med (n=3)

Beta diversity shown as principal component analysis (PCA) on genus-level hyperbolic arcsine transformed data. Ellipses indicate the 95% confidence interval.

C: HC (n=48), CVIDio –med (n=32), CVIDid –med (n=18), XLA-med (n=3). FDR-adjusted PERMANOVA CVIDio-med vs CVIDid-med p.adj=0.009. CVIDid vs HC p.adj=0.006

D: HC (n=48), CVID –med +IgA (n=29), CVID –med –IgA (n=21), XLA –med (n=3). FDR-adjusted PERMANOVA: CVID+IgA-med vs HC p.adj= 0.032, CVID-IgA-med vs HC p adj.=0.006


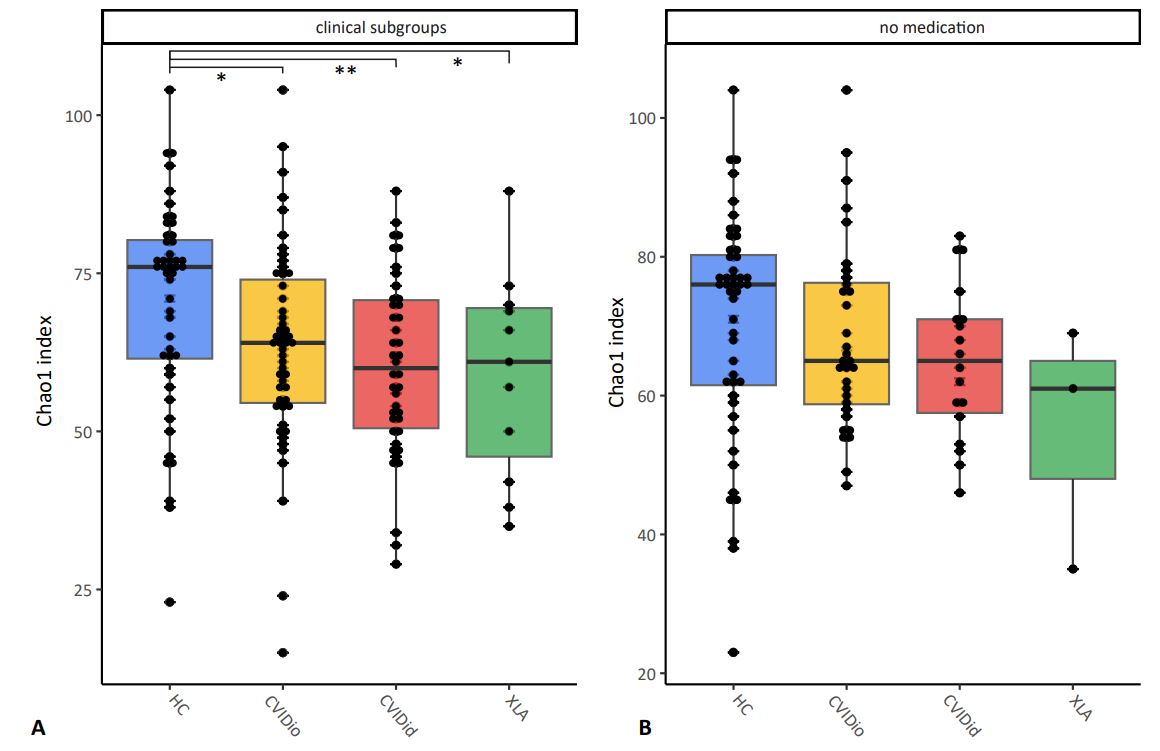

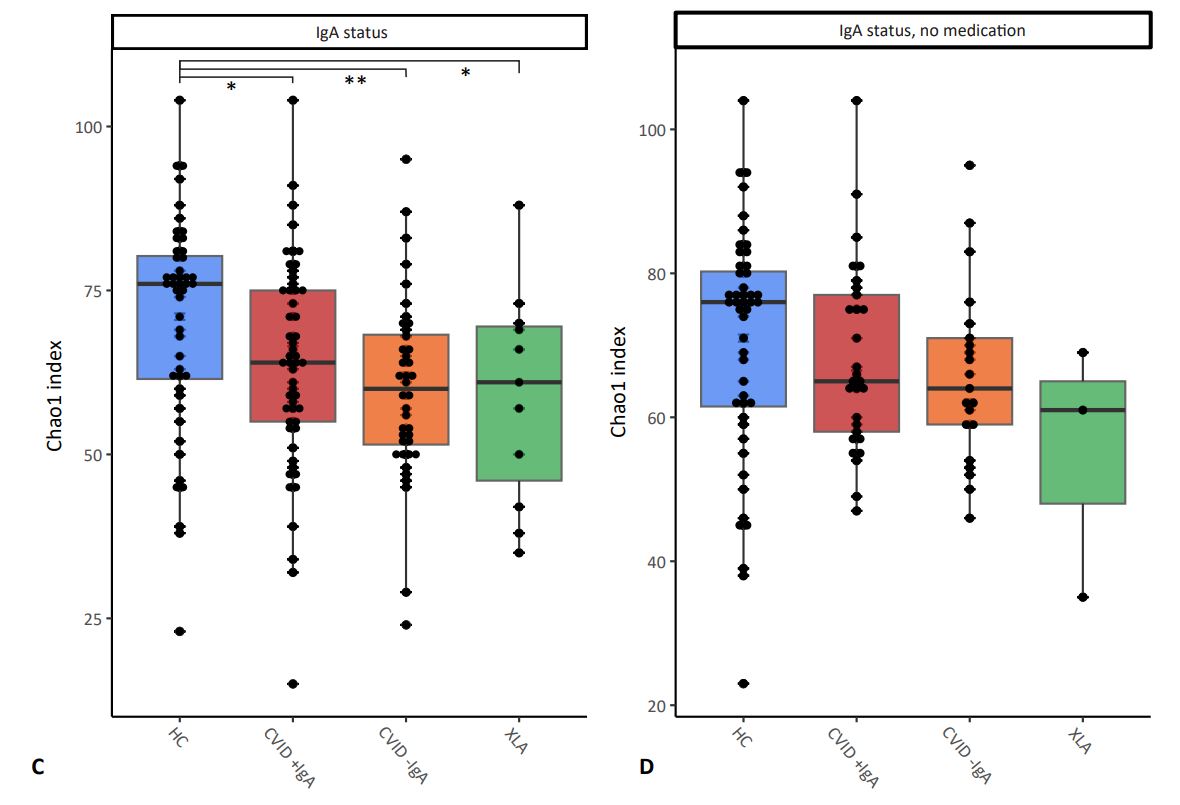


**Supplementary figure 4**: Richness as calculated using the Chao1 index. The horizontal line inside the box represents the median. The whiskers represent the lowest and highest values within 1.5 × interquartile range. P values boxplots: Mann–Whitney U test. * p<0.05, ** p<0.01, *** p<0.001.

A: Healthy controls (HC n=48), CVID with infections only (CVIDio, n=51) CVID with immune dysregulation (CVIDid, n=42), and X-linked agammaglobulinemia (XLA, n=11).

B: HC (n=48), CVIDio –med (n=32), CVIDid –med (n=18), XLA-med (n=3).

C: HC (n=48), CVID +IgA (n=53), CVID-IgA (n=40), XLA (n=11).

D: HC (n=48), CVID –med +IgA (n=29), CVID –med –IgA (n=21), XLA –med (n=3)


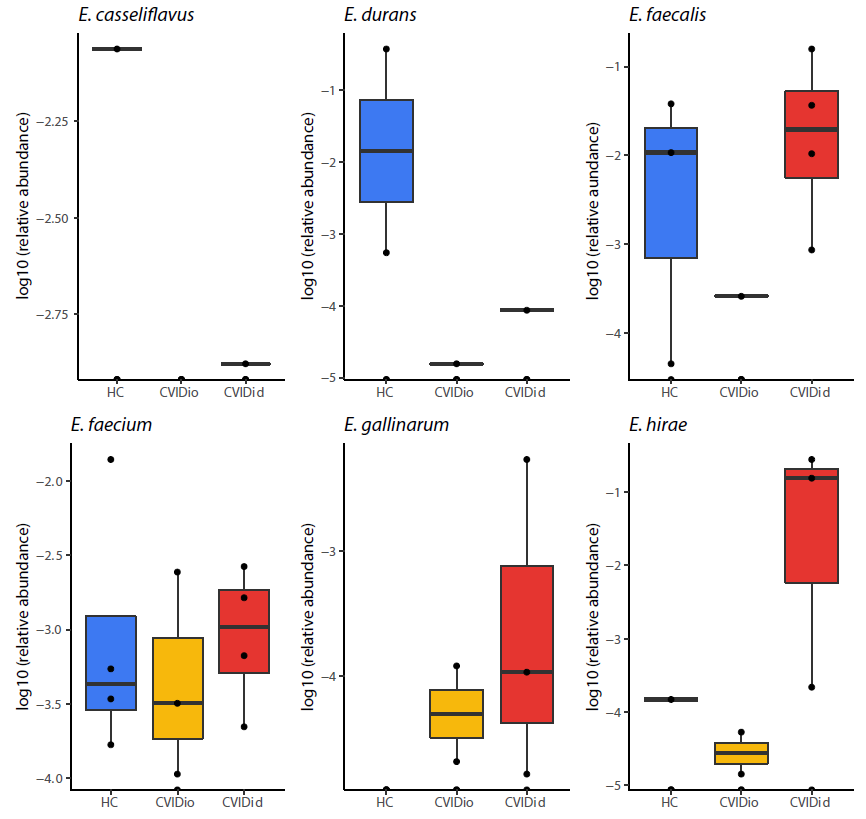


**Supplementary Figure 5**: Relative abundance of the most frequently detected enterococcal species determined using shotgun metagenomic sequencing in 4 HC, 4 CVIDio and 4 CVIDid. The horizontal line inside the box represents the median. The whiskers represent the lowest and highest values within 1.5 × interquartile range.


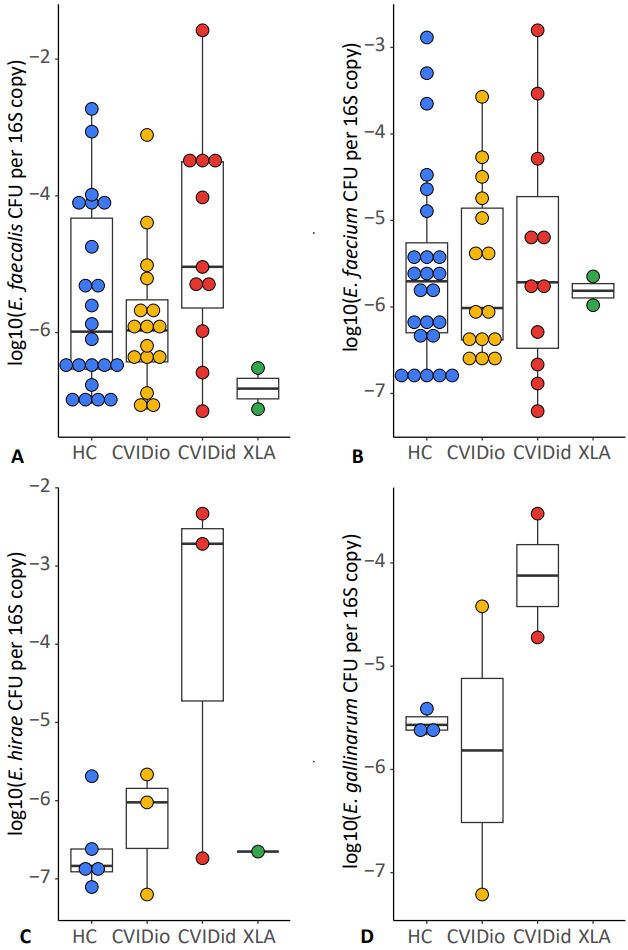


**Supplementary Figure 6**: Presence of enterococcal DNA in stools of controls and patients *without medication use*. This is expressed by the log 10 of the amount of enterococcal DNA present in one colony forming unit (CFU) corrected by the total 16S rRNA abundance per fecal sample as determined using qPCR. Results below the detection limit of the qPCR assay were not displayed. HC (HC, n=48), CVID with infections only (CVIDio, n=32), CVID with immune dysregulation (CVIDid, n=18), X-linked agammaglobulinemia (XLA, n=3).

The horizontal line inside the box represents the median. The whiskers represent the lowest and highest values within 1.5 × interquartile range. Statistics: Mann–Whitney U test. * p<0.05, ** p<0.01, *** p<0.001.


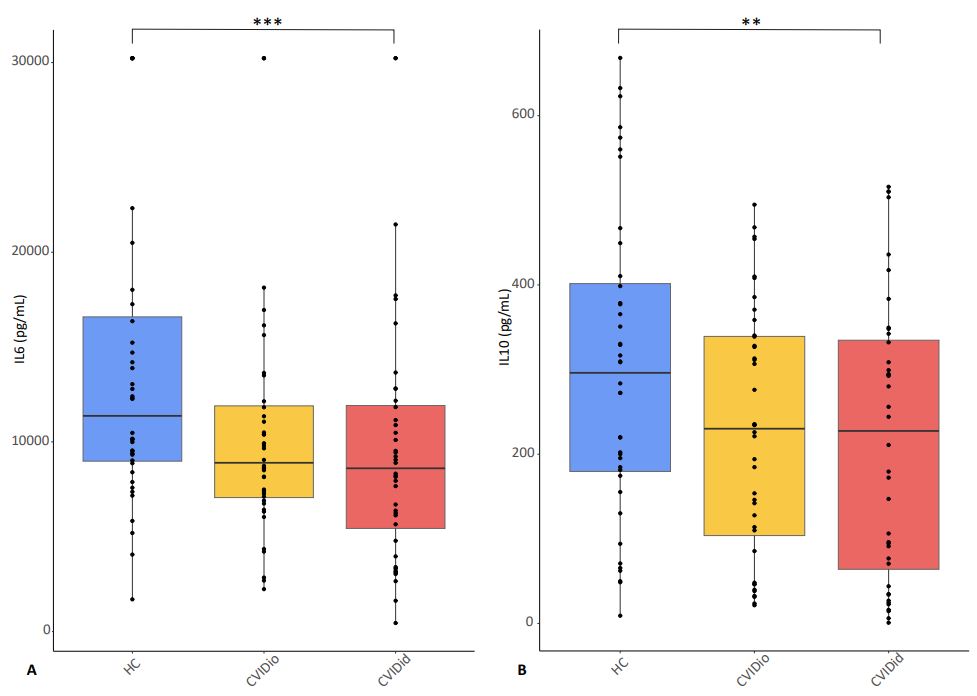

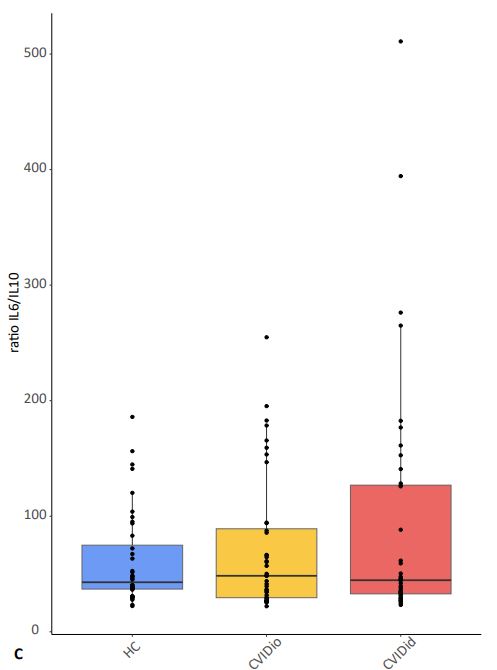


**Supplementary Figure 7:**

Cytokine production after *in-vitro* stimulation of monocytes from 10 HC, 10 CVIDio and 10 CVIDid with bacterial supernatants from *E.coli* E783, *E.gallinarum* E9950, *E.gallinarum* E.9951, *E.hirae* E9954 and *E.hirae* E9958 (pooled data). A: IL-6 production, B: IL-10 production, C: IL6/IL10 ratio.

The horizontal line inside the box represents the median. The whiskers represent the lowest and highest values within 1.5 × interquartile range. P values boxplots: Mann–Whitney U test. * p<0.05, ** p<0.01, *** p<0.001.

**Supplementary Table 1:** qPCR primer-probe sequences, ordered from IDT DNA technologies. All sequences reported from 5′-3’, all probes flanked by 6-FAM/ZEN (5’) and Iowa Black®Fluorescence Quencher^2^.

| BactQuant | |
| --- | --- |
| Target gene | 16S rRNA |
| Forward primer | CCTACGGGDGGCWGCA |
| Probe | CAGCAGCCGCGGTA |
| Reverse Primer | GGACTACHVGGGTMTCTAATC |
| *Enterococcus faecium* | |
| Target gene | *ddl* |
| Forward primer | TGCCTGGTGAAGTCGTAAAAG |
| Probe | CGAAATGCAGATTCCAGCCGAAGTG |
| Reverse Primer | AGCTAACTTCGCGTACTCTTG |
| *Enterococcus faecalis* | |
| target gene | *ddl* |
| Forward primer | GCACGTGAAATTGAAGTAGCC |
| Probe | TGGTGAAGTGGTGAAAGATGTCGCT |
| Reverse Primer | GAACATGCGCTGGGATTTG |
| *Enterococcus gallinarum* | |
| Target gene | *sodA* |
| Forward primer | TTTGATTCGGTGCCTGAAGA |
| Probe | AACGGTGGTGGTCATGCAAATCAC |
| Reverse Primer | AGCATTTGGTGCCAAGATTTC |
| *Enterococcus hirae* | |
| target gene | *ddl* |
| Forward primer | CAGTTCTTTCAGCGTTTTCAGTC |
| Probe | AGGGCCTTTCACCCATTGACCTTC |
| Reverse Primer | CTTTGCTCGTTGGTTTCTCTG |

**Supplementary Table 2:** Overview of all bacterial strains used in this study

| **Identifier** | **Species** | **Isolation source** | **Reference** | **Use in this study** |
| --- | --- | --- | --- | --- |
| E06019 | *E. faecium* | Stool hospitalized patient | Arredondo-Alonso et al.^3^ | Validation species-specific RT-PCR |
| E06007 | *E. faecium* | Stool hospitalized patient | Wagner et al.^4^ | Validation species-specific RT-PCR |
| E07089 | *E. gallinarum* | Stool hospitalized patient | In-house strain collection | Validation species-specific RT-PCR |
| E06960 | *E. gallinarum* | Stool hospitalized patient | In-house strain collection | Validation species-specific RT-PCR |
| E00277 | *E. hirae* | Stool human community | In-house strain collection | Validation species-specific RT-PCR |
| E00134 | *E. hirae* | Stool human community | In-house strain collection | Validation species-specific RT-PCR |
| E04246 | *E. faecalis* | Stool hospitalized patient | In-house strain collection | Validation species-specific RT-PCR |
| E04665 | *E. faecalis* | Stool hospitalized patient | In-house strain collection | Validation species-specific RT-PCR |
| E783 | *E. coli* | Bloodstream infection | In-house strain collection | Bacterial - immune cell cocultures |
| E09950 | *E. gallinarum* | Stool CVIDid | This study | Bacterial - immune cell cocultures, *fliC* analysis |
| E09951 | *E. gallinarum* | Stool CVIDid | This study | Bacterial - immune cell cocultures, *fliC* analysis |
| E09954 | *E. hirae* | Stool CVIDid | This study | Bacterial - immune cell cocultures, *fliC* analysis |
| E09958 | *E. hirae* | Stool CVIDid | This study | Bacterial - immune cell cocultures, *fliC* analysis |
| E09947 | *E. gallinarum* | Stool HC | This study | *fliC* analysis |
| E09946 | *E. gallinarum* | Stool CVIDio | This study | *fliC* analysis |
| E09948 | *E. gallinarum* | Stool HC | This study | *fliC* analysis |
| E09944 | *E. gallinarum* | Stool CVIDio | This study | *fliC* analysis |
| E09952 | *E. gallinarum* | Stool CVIDio | This study | *fliC* analysis |
| E09945 | *E. gallinarum* | Stool CVIDio | This study | *fliC* analysis |
| E09949 | *E. gallinarum* | Stool CVIDid | This study | *fliC* analysis |

**Supplementary Table 3:** Characteristics biopsy cohort.

|  | **HC** | **CVID** | **XLA** |
| --- | --- | --- | --- |
| **n** | 9 | 15 | 3 |
| **Age (years, median)** | 33 | 38 | 28 |
| **Sex (male)** | 2 (22%) | 6 (40%) | 3 (100%) |
| **Antibiotic use** | 0 | 7 (47%) | 0 |
| **Serum IgA < 0.1 g/L** | n.d. | 11 (73%) | 3 (100%) |
| **Histology colon biopsies** |  |  |  |
| No pathology | 9 | 4 (26%) | 1 (33%) |
| Lymphoid aggregates | 0 | 11 (73%) | 1 (33%) |
| Focal signs of chronic inflammation | 0 | 1 (7%) | 1 (33%) |
| Acute inflammation or infection | 0 | 0 | 0 |
| **Immune dysregulation symptoms** | 0 | 8 (53%) | 0 |
| Pulmonary | 0 | 3 (30%) | 0 |
| Hematological | 0 | 2 (13%) | 0 |
| Gastrointestinal | 0 | 2 (13%)# | 0 |
| Rheumatological | 0 | 1 (7%) | 0 |
| Lymphoproliferative | 0 | 7 (47%) | 0 |

#: both autoimmune gastritis**Supplementary table 4**: Differentially abundant bacteria identified using 16S rRNA sequencing. CVID (n=93) vs healthy control (HC, n=48): coefficients, standard errors (SE), p-values, FDR-adjusted p-values. Median relative abundance of non-zero counts per group. Statistics: ANCOM-BC.

| **Up in CVID** | **Beta CVID vs HC** | **SE CVID vs HC** | **p-value CVID vs HC** | **adj. p-value CVID vs HC** | **Beta age** | **SE age** | **p-value age** | **adj. p-value age** | **Beta sex** | **SE sex** | **p-value sex** | **adj. p-value sex** |
| --- | --- | --- | --- | --- | --- | --- | --- | --- | --- | --- | --- | --- |
| *Gammaproteobacteria* | -0.492 | 0.132 | 0 | 0.002 | 0.021 | 0.011 | 0.059 | 0.167 | 0.051 | 0.343 | 0.881 | 0.945 |
| *Enterobacteriaceae* | -0.462 | 0.132 | 0 | 0.007 | 0.018 | 0.012 | 0.128 | 0.441 | 0.184 | 0.381 | 0.628 | 0.94 |
| *Escherichia-Shigella* | -0.433 | 0.125 | 0.001 | 0.011 | 0.011 | 0.012 | 0.357 | 0.795 | -0.005 | 0.375 | 0.989 | 1 |
| *Bacilli* | -0.391 | 0.131 | 0.003 | 0.011 | 0.007 | 0.011 | 0.535 | 0.841 | -0.026 | 0.368 | 0.945 | 0.945 |
| *Enterobacteriales* | -0.372 | 0.135 | 0.006 | 0.025 | 0.017 | 0.012 | 0.139 | 0.334 | 0.125 | 0.377 | 0.74 | 0.962 |
| *Sellimonas* | -0.368 | 0.087 | 0 | 0 | -0.015 | 0.009 | 0.124 | 0.601 | 0.463 | 0.279 | 0.097 | 0.496 |
| *Proteobacteria* | -0.348 | 0.108 | 0.001 | 0.004 | 0.025 | 0.01 | 0.013 | 0.038 | 0.067 | 0.311 | 0.828 | 0.828 |
| *Ruminococcus 1* | -0.336 | 0.12 | 0.005 | 0.035 | -0.002 | 0.013 | 0.879 | 0.944 | 0.14 | 0.394 | 0.722 | 0.911 |
| *Ruminococcaceae UCG-004* | -0.311 | 0.107 | 0.004 | 0.035 | -0.011 | 0.009 | 0.208 | 0.756 | 0.666 | 0.293 | 0.023 | 0.204 |
| *Eggerthella* | -0.272 | 0.089 | 0.002 | 0.034 | -0.015 | 0.008 | 0.068 | 0.531 | 0.786 | 0.267 | 0.003 | 0.116 |
| *Alloprevotella* | -0.25 | 0.07 | 0 | 0 | 0.004 | 0.008 | 0.586 | 0.846 | -0.186 | 0.23 | 0.42 | 0.789 |
| *Flavonifractor* | -0.248 | 0.084 | 0.003 | 0.035 | -0.006 | 0.008 | 0.415 | 0.795 | 0.4 | 0.253 | 0.113 | 0.524 |
| *Erysipelatoclostridium* | -0.247 | 0.068 | 0 | 0.01 | 0 | 0.007 | 0.94 | 0.968 | 0.116 | 0.215 | 0.589 | 0.897 |
| *Oscillibacter* | -0.233 | 0.088 | 0.008 | 0.048 | -0.004 | 0.008 | 0.644 | 0.846 | -0.043 | 0.255 | 0.865 | 0.983 |
| *Tyzzerella* | -0.231 | 0.078 | 0 | 0 | 0.003 | 0.007 | 0.673 | 0.858 | 0.072 | 0.238 | 0.762 | 0.915 |
| *Tyzzerella 3* | -0.229 | 0.082 | 0.005 | 0.035 | -0.002 | 0.007 | 0.839 | 0.934 | -0.203 | 0.262 | 0.439 | 0.789 |
| *Parasutterella* | -0.225 | 0.08 | 0.005 | 0.035 | -0.006 | 0.007 | 0.415 | 0.795 | 0.328 | 0.239 | 0.169 | 0.669 |
| *Veillonella* | -0.22 | 0.09 | 0 | 0 | -0.005 | 0.011 | 0.621 | 0.846 | -0.197 | 0.308 | 0.521 | 0.858 |
| *Blautia* | -0.218 | 0.068 | 0.001 | 0.023 | -0.005 | 0.005 | 0.399 | 0.795 | 0.447 | 0.176 | 0.011 | 0.187 |
| *Firmicutes* | -0.213 | 0.06 | 0 | 0.002 | 0 | 0.005 | 0.935 | 0.935 | 0.094 | 0.17 | 0.578 | 0.828 |
| *Clostridia* | -0.199 | 0.06 | 0.001 | 0.005 | -0.001 | 0.005 | 0.792 | 0.882 | 0.091 | 0.151 | 0.55 | 0.945 |
| *Eubacterium 1* | -0.192 | 0.067 | 0.004 | 0.035 | -0.002 | 0.006 | 0.784 | 0.93 | 0.197 | 0.197 | 0.315 | 0.765 |
| *Lactococcus* | -0.148 | 0.053 | 0 | 0 | -0.01 | 0.005 | 0.043 | 0.522 | 0.221 | 0.167 | 0.187 | 0.699 |
| *Eubacteriaceae* | -0.145 | 0.054 | 0 | 0 | -0.002 | 0.005 | 0.762 | 0.909 | 0.371 | 0.162 | 0.022 | 0.209 |
| *Caproiciproducens* | -0.137 | 0.053 | 0 | 0 | -0.014 | 0.005 | 0.006 | 0.147 | 0.16 | 0.154 | 0.299 | 0.765 |
| **Up in HC** |  |  |  |  |  |  |  |  |  |  |  |  |
| *Bifidobacteriales* | 0.522 | 0.125 | 0 | 0 | -0.041 | 0.012 | 0 | 0.006 | -0.562 | 0.379 | 0.138 | 0.897 |
| *Bifidobacterium* | 0.433 | 0.121 | 0 | 0.01 | -0.045 | 0.012 | 0 | 0.008 | -0.554 | 0.382 | 0.147 | 0.653 |
| *Bifidobacteriaceae* | 0.431 | 0.121 | 0 | 0.007 | -0.04 | 0.012 | 0.001 | 0.02 | -0.503 | 0.379 | 0.185 | 0.521 |
| *Prevotella 9* | 0.383 | 0.145 | 0.008 | 0.048 | -0.017 | 0.01 | 0.098 | 0.595 | -0.485 | 0.387 | 0.21 | 0.699 |
| *Romboutsia* | 0.346 | 0.124 | 0.005 | 0.035 | -0.04 | 0.01 | 0.001 | 0.005 | 0.436 | 0.367 | 0.235 | 0.699 |
| *Actinobacteria* | 0.312 | 0.131 | 0.017 | 0.048 | -0.041 | 0.012 | 0.001 | 0.006 | -0.555 | 0.377 | 0.141 | 0.737 |
| *Selenomonadales* | 0.269 | 0.098 | 0.006 | 0.025 | -0.002 | 0.008 | 0.8 | 0.876 | -0.358 | 0.27 | 0.186 | 0.897 |

**Supplementary Table 5:** Differentially abundant bacteria identified using 16S rRNA sequencing. CVID without use of antibiotics or immunosuppressive therapy 3 months prior to sampling (CVID-med n=50) vs healthy control (HC. n=48): coefficients. standard errors (SE), p-values, FDR-adjusted p-values. Median relative abundance of non-zero counts per group. Statistics: ANCOM-BC.

| **Up in CVID-med** | **Beta CVID-med vs HC** | **SE CVID-med vs HC** | **p-value CVID-med vs HC** | **adj. p-value CVID-med vs HC** | **Beta age** | **SE age** | **p-value age** | **adj. p-value age** | **Beta sex** | **SE sex** | **p-value sex** | **adj. p-value sex** |
| --- | --- | --- | --- | --- | --- | --- | --- | --- | --- | --- | --- | --- |
| *Eubacteriaceae* | -0.156 | 0.067 | 0.000 | 0.000 | -0.003 | 0.006 | 0.687 | 0.852 | 0.369 | 0.205 | 0.072 | 0.439 |
| *Lactococcus* | -0.138 | 0.058 | 0.000 | 0.000 | -0.012 | 0.005 | 0.008 | 0.175 | -0.019 | 0.171 | 0.912 | 0.936 |
| *Sellimonas* | -0.301 | 0.089 | 0.000 | 0.000 | -0.016 | 0.009 | 0.068 | 0.445 | -0.120 | 0.290 | 0.678 | 0.893 |
| *Tyzzerella* | -0.197 | 0.085 | 0.000 | 0.000 | 0.002 | 0.008 | 0.830 | 0.974 | -0.444 | 0.251 | 0.077 | 0.332 |
| *Anaerotruncus* | -0.152 | 0.062 | 0.000 | 0.000 | -0.001 | 0.005 | 0.804 | 0.965 | -0.039 | 0.172 | 0.820 | 0.913 |
| *Caproiciproducens* | -0.119 | 0.059 | 0.000 | 0.000 | -0.021 | 0.006 | 0.000 | 0.016 | -0.166 | 0.180 | 0.356 | 0.674 |
| *Veillonella* | -0.204 | 0.110 | 0.000 | 0.000 | -0.012 | 0.012 | 0.300 | 0.727 | -0.577 | 0.353 | 0.102 | 0.401 |

**Supplementary Table 6**: Differentially abundant bacteria identified using 16S rRNA sequencing. CVID with immune dysregulation (CVIDid. n=42) versus CVID with infections only (CVIDio. n =51): coefficients. standard errors (SE), p-values, FDR-adjusted p-values. Median relative abundance of non-zero counts per group. Statistics: ANCOM-BC.

| **Up in CVIDid** | **Beta CVIDid - CVIDio** | **SE CVIDid - CVIDio** | **p-value CVIDid - CVIDio** | **adj. p-value CVIDid - CVIDio** | **Beta age** | **SE age** | **p-value age** | **adj. p-value age** | **Beta sex** | **SE sex** | **p-value sex** | **adj. p-value sex** |
| --- | --- | --- | --- | --- | --- | --- | --- | --- | --- | --- | --- | --- |
| Enterococcaceae | -0.811 | 0.296 | 0 | 0 | -0.003 | 0.008 | 0.71 | 0.928 | 0.121 | 0.279 | 0.664 | 0.981 |
| *Enterococcus* | -0.919 | 0.297 | 0 | 0 | -0.004 | 0.008 | 0.632 | 0.977 | -0.022 | 0.274 | 0.937 | 0.963 |

**Supplementary Table 7:**

Differentially abundant bacteria identified using 16S rRNA sequencing. CVID with immune dysregulation without medication use (CVIDid -med. n=18) versus CVID with infections only without medication use (CVIDio -med. n=32). Patients did not use antibiotics or immunosuppressive therapy 3 months prior to sampling: coefficients. standard errors (SE), p-values, FDR-adjusted p-values. Median relative abundance of non-zero counts per group. Statistics: ANCOM-BC.

| **Up in CVIDid-med** | **Beta CVIDid med vs CVIDio -med** | **SE CVIDid med vs CVIDio -med** | **p-value CVIDid med vs CVIDio -med** | **adj. p-value CVIDid med vs CVIDio -med** | **Beta age** | **SE age** | **p-value age** | **adj. p-value age** | **Beta sex** | **SE sex** | **p-value sex** | **adj. p-value sex** |
| --- | --- | --- | --- | --- | --- | --- | --- | --- | --- | --- | --- | --- |
| *Enterococcaceae* | -0.998 | 0.407 | 0.000 | 0.000 | -0.011 | 0.011 | 0.305 | 0.840 | -0.082 | 0.365 | 0.823 | 0.933 |
| *Peptococcaceae* | -0.039 | 0.258 | 0.000 | 0.000 | 0.008 | 0.008 | 0.332 | 0.840 | 0.221 | 0.249 | 0.375 | 0.708 |
| *uncultured.2* | -0.085 | 0.278 | 0.000 | 0.000 | 0.004 | 0.007 | 0.607 | 0.840 | -0.266 | 0.274 | 0.330 | 0.661 |
| *Pasteurellaceae* | -0.269 | 0.303 | 0.000 | 0.000 | -0.005 | 0.008 | 0.545 | 0.840 | -0.181 | 0.252 | 0.473 | 0.759 |
| *Rhodospirillales* | -0.016 | 0.297 | 0.000 | 0.000 | 0.005 | 0.008 | 0.487 | 0.852 | -0.052 | 0.284 | 0.854 | 0.903 |
| *Pasteurellales* | -0.200 | 0.320 | 0.000 | 0.000 | -0.003 | 0.009 | 0.708 | 0.905 | 0.033 | 0.270 | 0.903 | 0.903 |
| *Alphaproteobacteria* | -0.053 | 0.293 | 0.000 | 0.000 | 0.004 | 0.007 | 0.555 | 0.879 | -0.265 | 0.280 | 0.344 | 0.825 |
| *Enterococcus* | -0.102 | 0.431 | 0.000 | 0.000 | -0.013 | 0.011 | 0.245 | 0.792 | -0.468 | 0.373 | 0.210 | 0.440 |
| *Eubacterium 2* | -0.243 | 0.388 | 0.000 | 0.000 | 0.009 | 0.009 | 0.303 | 0.836 | -0.204 | 0.380 | 0.591 | 0.776 |
| *Anaerotruncus* | -0.192 | 0.273 | 0.000 | 0.000 | 0.003 | 0.006 | 0.654 | 0.907 | -0.117 | 0.261 | 0.653 | 0.816 |
| *Veillonella* | -0.122 | 0.574 | 0.000 | 0.000 | -0.013 | 0.017 | 0.461 | 0.880 | -0.559 | 0.553 | 0.312 | 0.537 |
| *gut metagenome.1* | -0.101 | 0.246 | 0.000 | 0.000 | 0.002 | 0.006 | 0.739 | 0.919 | -0.653 | 0.237 | 0.006 | 0.066 |
| *Haemophilus* | -0.286 | 0.296 | 0.000 | 0.000 | -0.007 | 0.008 | 0.393 | 0.880 | -0.568 | 0.231 | 0.014 | 0.082 |

**Supplementary Table 8:**

Differentially abundant bacteria identified using 16S rRNA sequencing. CVID with IgA <0.1 g/L (CVID-IgA n=40) versus CVID with IgA >0.1g/L (CVID+IgA n=53): coefficients. standard errors (SE). p-values. FDR-adjusted p-values. Median relative abundance of non-zero counts per group. Statistics: ANCOM-BC.

| **Up in CVID-IgA** | **Beta CVID-IgA - CVID+IgA** | **SE CVID-IgA - CVID+IgA** | **p-value CVID-IgA - CVID+IgA** | **adj. p-value CVID-IgA - CVID+IgA** | **Beta age** | **SE age** | **p-value age** | **adj. p-value age** | **Beta sex** | **SE sex** | **p-value sex** | **adj. p-value sex** |
| --- | --- | --- | --- | --- | --- | --- | --- | --- | --- | --- | --- | --- |
| *Enterococcaceae* | -0.471 | 0.303 | 0 | 0 | -0.003 | 0.008 | 0.670 | 0.938 | 0.090 | 0.286 | 0.753 | 0.988 |
| *Enterococcus* | -0.595 | 0.3057 | 0 | 0 | -0.004 | 0.008 | 0.577 | 0.982 | -0.049 | 0.284 | 0.863 | 0.927 |
| *Eubacterium 2* | -0.866 | 0.328 | 0 | 0 | 0.004 | 0.007 | 0.633 | 0.982 | 0.152 | 0.294 | 0.605 | 0.816 |

**Supplementary Table 9:**

Differentially abundant bacteria identified using 16S rRNA sequencing. CVID with IgA <0.1 g/L without medication use (CVID-IgA-med n=21) versus CVID with IgA >0.1g/L without medication use (CVID+IgA-med n=29): coefficients. standard errors (SE). p-values. FDR-adjusted p-values. Median relative abundance of non-zero counts per group. Statistics: ANCOM-BC.

| **up in CVID-IgA-med** | **Beta CVID-IgA-med vs CVID+IgA-med** | **SE CVID-IgA-med vs CVID+IgA-med** | **p-value CVID-IgA-med vs CVID+IgA-med** | **adj. p-value CVID-IgA-med vs CVID+IgA-med** | **Beta age** | **SE age** | **p-value age** | **adj. p-value age** | **Beta sex** | **SE sex** | **p-value sex** | **adj. p-value sex** |
| --- | --- | --- | --- | --- | --- | --- | --- | --- | --- | --- | --- | --- |
| *Veillonella* | -0.116 | 0.609 | 0.000 | 0.000 | -0.019 | 0.019 | 0.313 | 0.933 | -0.513 | 0.588 | 0.383 | 0.629 |
| *Eubacterium 2* | -0.658 | 0.389 | 0.000 | 0.000 | 0.004 | 0.009 | 0.629 | 0.933 | -0.140 | 0.360 | 0.698 | 0.890 |
| *Haemophilus* | -0.531 | 0.297 | 0.000 | 0.000 | -0.010 | 0.008 | 0.173 | 0.933 | -0.523 | 0.237 | 0.028 | 0.138 |
| *Enterococcus* | -0.489 | 0.458 | 0.000 | 0.000 | -0.015 | 0.014 | 0.258 | 0.933 | -0.489 | 0.392 | 0.212 | 0.464 |
| *Pasteurellaceae* | -0.401 | 0.301 | 0.000 | 0.000 | -0.005 | 0.008 | 0.476 | 0.984 | -0.189 | 0.248 | 0.446 | 0.738 |
| *Pasteurellales* | -0.364 | 0.312 | 0.000 | 0.000 | -0.004 | 0.008 | 0.651 | 0.911 | 0.034 | 0.268 | 0.898 | 0.898 |
| *Enterococcaceae* | -0.359 | 0.431 | 0.000 | 0.000 | -0.010 | 0.013 | 0.417 | 0.984 | -0.156 | 0.378 | 0.680 | 0.878 |

**Supplementary Table 10:** Baseline characteristics of the matched serum-stool cohort.

| **Summary statistics matched serum-stool cohort** | **HC (N=31)** | **CVIDio (N=29)** | **CVIDid (N=28)** |
| --- | --- | --- | --- |
| **Characteristics** |  |  |  |
| Sex (male) | 10 (32.3%) | 13 (44.8%) | 17 (60.7%) |
| Age - Median [Min. Max] | 41.0 [20.0. 69.0] | 40.0 [18.0. 68.0] | 39.5 [12.0. 68.0] |
| Antibiotics | 0 (0%) | 6 (20.7%) | 9 (32.1%) |
| Immunosuppressive medication | 0 (0%) | 0 (0%) | 2 (7.1%) |
| Serum IgA >0.1g/L | 31 (100%) | 19 (65.5%) | 10 (35.7%) |
| **Immune dysregulation complications** |  |  |  |
| Pulmonary | 0 (0%) | 0 (0%) | 9 (32.1%) |
| Hematological | 0 (0%) | 0 (0%) | 6 (21.4%) |
| Gastrointestinal | 0 (0%) | 0 (0%) | 14 (50.0%) |
| Rheumatological | 0 (0%) | 0 (0%) | 5 (17.9%) |
| Dermatological | 0 (0%) | 0 (0%) | 5 (17.9%) |
| Lymphoproliferative | 0 (0%) | 0 (0%) | 13 (46.4%) |
| Other | 0 (0%) | 0 (0%) | 1 (3.6%) |
| **Genetics** |  |  |  |
| Genetics not done | 31 (100%) | 28 (96.6%) | 21 (75.0%) |
| Nothing found | 0 (0%) | 0 (0%) | 2 (7.1%) |
| Only VUS found | 0 (0%) | 0 (0%) | 2 (7.1%) |
| Pathogenic mutations found | 0 (0%) | 0 (0%) | 3 (10.7%) |

**References Supplementary Material**

1. Lauté-Caly. D. L. *et al.* The flagellin of candidate live biotherapeutic Enterococcus gallinarum MRx0518 is a potent immunostimulant. *Sci. Rep.* **9**. 1–14 (2019).

2. Liu. C. M. *et al.* BactQuant : An enhanced broad-coverage bacterial quantitative real-time PCR assay. *BMC Microbiol.* **12**. 56 (2012).

3. Arredondo-Alonso. S. *et al.* Plasmids shaped the recent emergence of the major nosocomial pathogen Enterococcus faecium. *MBio* **11**. 1–17 (2020).

4. Wagner. T. M. *et al.* Enterococcus faecium TIR-Domain Genes Are Part of a Gene Cluster Which Promotes Bacterial Survival in Blood. *Int. J. Microbiol.* 2018).
